# Population attributable fractions of modifiable dementia risk factors in the Netherlands: a cross-sectional, time series analysis

**DOI:** 10.1016/j.eclinm.2026.104018

**Published:** 2026-06-16

**Authors:** Tessa L. van Baal, Niels Janssen, Lukas A. Duffner, Sebastian Köhler, Kay Deckers

**Affiliations:** a Alzheimer Centre Limburg, Department of Psychiatry and Neuropsychology, Mental Health and Neuroscience Research Institute (MHeNs), Maastricht University, Maastricht, the Netherlands; b Alzheimer Europe, Senningerberg, Luxembourg

**Keywords:** Dementia, Risk reduction, Population attributable fractions, Health equity, The Netherlands

## Abstract

**Background:**

Approximately 310,000 individuals in the Netherlands are currently living with dementia, and this number is projected to double by 2050. Examining the distribution of modifiable risk and protective factors is essential for developing effective dementia risk reduction strategies. This study aimed to estimate the population attributable fractions (PAFs) of seven common modifiable risk factors for dementia in the Netherlands.

**Methods:**

Nationally representative cross-sectional data from four waves of the Dutch Public Health Monitor surveys (2012–2022) were analysed, with sample sizes ranging from 302,422 to 409,278 respondents. Individuals were included in the analytical sample if data were available on all seven modifiable risk factors for dementia. PAFs were calculated using a life-course approach, categorising low educational attainment in earlylife (<45 years); obesity, physical inactivity, smoking, excessive alcohol consumption, and depression in mid-life (45–65 years); and social isolation in late-life (>65 years). PAFs were estimated overall and stratified by sex, household income, migration background, and region.

**Findings:**

Overall PAFs remained stable, with estimates of 30·9% [16·8–43·4] in 2012, 29·9% [16·4–42·1] in 2016, 29·7% [16·5–41·4] in 2020, and 31·1% [17·7–43·2] in 2022. The largest proportion of dementia cases was attributable to social isolation, followed by physical inactivity and depression. Stratified analyses showed comparable PAFs between females and males. In contrast, differences were observed across other subgroups. PAFs were higher among people with lower household income and among first-generation migrants. PAFs also showed regional variation.

**Interpretation:**

Despite shifts in individual modifiable risk factors, the overall potential for dementia risk reduction in the Netherlands remained stable over the past decade. At the same time, marked inequalities persisted across regions and structurally disadvantaged groups. Future work should prioritise the development of targeted, equity-focused dementia risk reduction strategies.

**Funding:**

The BIRD-NL consortium, funded by The Netherlands Organisation for Health Research and Development (ZonMw) as part of the National Dementia Strategy 2021–2030 of the Ministry of Health, Welfare and Sport.

**Research in context**

**Evidence before this study**

A literature search was conducted in PubMed and Google Scholar for articles published before July 8, 2025, using the terms (“attributable fraction” OR “prevention” OR “PAF”) AND (“dementia”). Search terms were restricted to titles and abstracts, with no language restrictions. A 2024 systematic review and meta-analysis identified population-attributable fraction (PAF) estimates for dementia across 25 countries. These studies varied widely in the number and definition of modifiable risk factors included, with reported PAFs ranging from 24·4% to 66·8%, highlighting substantial between-country variability and the importance of context-specific estimates. To date, no study has examined temporal changes in dementia PAFs in the Netherlands or assessed modifiable dementia risk stratified by sex, household income, migration background, and region within the Dutch population.

**Added value of this study**

This study provides estimates of dementia PAFs in the Netherlands over a 10-year period using seven modifiable risk factors. It adds to existing evidence by examining variation in dementia risk across sex, household income, migration background, and region.

**Implications of all the available evidence**

Declines in the PAFs of modifiable risk factors, including smoking, low educational attainment, and excessive alcohol consumption, were offset by increases in depression, physical inactivity, obesity, and social isolation, resulting in stable dementia PAFs over time. Unequal distributions of PAFs across sociodemographic groups emphasise the need for risk reduction approaches that are both targeted and equity-focused.

## Introduction

In line with global trends, dementia is expected to become one of the most significant public health challenges facing the Netherlands in the coming decades.^1^ This projected increase is primarily driven by an ageing population, with the number of people living with dementia estimated to rise from 310,000^2^ in 2025 to 520,000 by 2040.^3^ Consequently, dementia is anticipated to become the leading cause of disease burden in the Netherlands,^4^ with significant implications for affected individuals, their caregivers, and society as a whole. The economic impact in the Netherlands is expected to be substantial, with dementia-related healthcare costs projected to increase by 135%, from €6·6 billion in 2015 (7·0% of total healthcare expenditure) to €15·6 billion by 2040.^4^

Efforts to mitigate the impact of dementia have focused on two complementary approaches: the development of disease-modifying therapies and lifestyle-based dementia risk reduction strategies. However, despite ongoing advances in next-generation pharmacological treatments, concerns persist regarding their efficacy, cost-effectiveness, safety, and limited applicability.^5^ Dementia risk reduction is, therefore, gaining prominence as a key strategy for addressing modifiable risk factors throughout the life course.

The 2024 report of the Lancet Commission on Dementia Prevention, Intervention, and Care used the most recent global data on the prevalence and relative risk of various factors to calculate population attributable fractions (PAFs) for dementia, which estimates the proportion of dementia cases that may be attributable to specific risk factors under modelling assumptions, including the hypothetical elimination of these exposures.^5^ They identified 14 modifiable dementia risk factors: low educational attainment, hearing loss, hypertension, smoking, obesity, depression, physical inactivity, diabetes, excessive alcohol consumption, traumatic brain injury, air pollution, social isolation, untreated vision loss, and elevated low-density lipoprotein cholesterol. Collectively, these risk factors have been estimated to account for approximately 45% of dementia cases worldwide.^5^

Although the complete elimination of modifiable risk factors is unlikely to be feasible, PAFs may be useful for assessing the potential population burden of dementia attributable to modifiable risk factors and examining the distribution of these risk factors within and between groups and regions. However, a global PAF estimate may not translate uniformly across countries. A systematic review and meta-analysis of 74 studies found cross-country variability in PAF estimates for all-cause dementia, with higher estimates in low- and middle-income countries than in high-income countries.^6^ Reported estimates ranged from 24·4% to 66·8%.^6^ This variability may reflect differences in the prevalence of modifiable risk factors, as well as methodological differences between studies, including study design, and the number and operationalisation of risk factors.^6^ Given these disparities, calculating country-specific PAFs is essential to ensure contextual accuracy and reflect each population's unique demographic, cultural, socioeconomic, political, and geographic characteristics.

However, even country-specific PAF estimates may obscure variation within populations. For example, individuals with a lower socioeconomic position appear to be at a higher risk of developing dementia,^7^ and females may be disproportionately affected, potentially due to social inequalities and sex differences, including reproductive factors and hormonal influences.^8^^,^^9^ Moreover, in a Dutch multiethnic prospective cohort study, dementia risk scores were found to be higher among migrant populations than among Dutch natives.^10^ Given the Netherlands' heterogeneous population, including numerous small migrant communities and marked interregional (health) inequalities,^11^ addressing within-country disparities in dementia risk is of importance.

Furthermore, monitoring dementia risk over time is essential for assessing how policy changes, sociodemographic shifts, or external events such as the Coronavirus disease (COVID-19) pandemic may influence modifiable risk factors.

To address this gap, this study aims to estimate cross-sectionally derived PAFs for seven modifiable dementia risk factors in the Netherlands for the years 2012, 2016, 2020, and 2022, stratified by sex, household income, migration background, and region.

## Methods

Data were utilised from the Dutch Public Health Monitor surveys^12^ conducted in 2012 (n = 387,195), 2016 (n = 457,153), 2020 (n = 539,902), and 2022 (n = 364,557) in adults aged 18 years and older by 25 Municipal Public Health Services (Gemeentelijke Gezondheidsdienst, GGD) across the Netherlands. These nationwide surveys are conducted every four years in collaboration with the GGDs, the National Institute for Public Health and the Environment (Rijksinstituut voor Volksgezondheid en Milieu), and Statistics Netherlands (Centraal Bureau voor de Statistiek, CBS); however, an additional round took place in 2022 due to the COVID-19 pandemic.

To ensure a representative sample of the population in the Netherlands for each survey year, respondents were selected using a complex sampling design from a stratified sample of non-institutionalised adults aged 18 years and older, based on region and age category.

A mixed-mode design was used: individuals were sent surveys that were primarily completed online, but surveys were also available in paper format, by telephone, or in person. Surveys were offered in Dutch, English, Turkish, and Moroccan Arabic. Data collection was conducted during the final four months of each survey year.

### Ethics

Following the Dutch Medical Research Involving Human Subjects Act (WMO), formal ethical approval was not required for this study, as it involved the analysis of existing anonymised survey data and did not subject respondents to procedures or interventions. All respondents provided informed consent by voluntarily completing the Public Health Monitor survey. Access to the datasets was granted through the official governance procedures of the national umbrella organisation of the GGDs and Regional Medical Assistance Organisations (Gemeentelijke Gezondheidsdienst voor de Geneeskundige Hulpverleningsorganisatie in de Regio, GGD GHOR Nederland) and CBS.

### Sociodemographics

CBS obtained respondents sociodemographic data from the Personal Records Database (Basisregistratie Personen), including data such as age (continuous in years and categories^5^: early-life (<45 years); mid-life (45–65 years); late-life (>65 years)), sex (male or female), and migration background (Dutch origin, first-generation migrants, or second-generation migrants). Household income quantiles were determined by CBS using registry-based national income distributions from the preceding year, adjusted for household size and composition. An exception was the 2012 Public Health Monitor survey, for which income quantiles were based on 2010 national income data (Table S1). Self-reported education was classified into three levels: (1) low (no education, primary education, pre-vocational secondary education, and junior secondary vocational education); (2) middle (senior general secondary education, pre-university secondary education, and secondary vocational education); (3) high (higher vocational education, and university). Marital status was also self-reported and categorised as: married/cohabiting, single/never been married, divorced, and widowed. Twenty-five regions in the Netherlands were defined according to the GGD classification system (Figure S1).

### Modifiable risk factors of dementia

Previously established modifiable risk factors were included to estimate the proportion of dementia cases attributable to modifiable risk factors.^5^ For the present analysis, data were available for seven of the 14 established modifiable dementia risk factors: low educational attainment, obesity, physical inactivity, excessive alcohol consumption, smoking, social isolation, and depression. The operationalisations of each self-reported risk factor and their availability are detailed in Panel 1.

Panel 1 **Operationalisations of modifiable dementia risk factors.**

**Table:** 

Risk factors	Operationalisation
**Early-life (<45 years)**	
Low educational attainment	No education; or primary education, pre-vocational secondary education, junior secondary vocational education.
**Mid-life (45–65 years)**	
Obesity	Body mass index (BMI) ≥30 kg/m^2^.
Physical inactivity	Nonadherence to the recommended guidelines of ≥150 min of moderate to vigorous physical activity minutes per week.^13^
Excessive alcohol consumption	Alcohol consumption exceeding 7 standard glasses per week.
Smoking	Current smoker.
Depression	Kessler Psychological Distress Scale (K10) questionnaire score of 30–50.^14^
**Late-life (>65 years)**	
Social isolation	Data on social isolation was unavailable. De Jong Gierveld Loneliness Scale score of ≥3^15^ was used as a proxy.

### Statistical analysis

Sampling weights for each Public Health Monitor survey year were calculated by CBS using a generalised linear model that incorporated sociodemographic and regional characteristics variables (e.g., age, urbanicity, sex, country of origin, and marital status) and their interactions.^16^, ^17^, ^18^, ^19^ Survey-specific sampling weights were applied to all sociodemographic characteristics and prevalence estimates of modifiable dementia risk factors to improve the sample's representativeness of the population in the Netherlands. Descriptive statistics were summarised as frequencies (percentages) or means ± standard deviation (SD) for both weighted and unweighted estimates.

Analyses were restricted to respondents with complete data on all seven modifiable dementia risk factors.

The formulae for calculating PAFs are presented in Panel 2. The prevalence of each modifiable dementia risk factor was weighted using the survey-specific sampling weights and grouped by life-course stage (early-life: <45 years; mid-life: 45–65 years; late-life: >65 years). The relative risks (RR) were obtained from the 2024 report of the Lancet Commission on Dementia Prevention, Intervention, and Care, which derived these estimates through a systematic review and meta-analyses.^5^ To account for shared variance among the risk factors and prevent overestimation of the cumulative PAF, communalities were calculated.^5^ First, tetrachoric correlation matrices were calculated using the seven dichotomised risk factors for each survey year. A principal component analysis was then performed on the correlation matrices, retaining components with eigenvalues ≥1. Communalities were subsequently calculated by summing the squared factor loadings, yielding survey-year-specific estimates for each risk factor. PAF 95% confidence intervals (CI) were calculated using the upper and lower bounds of the RR 95% CI in Levin's PAF formula, as previously described.^6^

Panel 2 **Calculation of population attributable fractions and communalities of modifiable dementia risk factors.**

**Table:** 

Step 1	**The PAFs of individual risk factors** were calculated using the following formula: $PAF=\frac{P_{e\;}\left({RR}_{e\;}-1\right)}{1+P_{e\;}\left({RR}_{e\;}-1\right)}$ where P_e_ represents the prevalence of the exposure, and RR_e_ denotes the relative risk of disease (dementia) associated with that exposure.
Step 2	**Communalities for each risk factor** were calculated by first deriving a tetrachoric correlation matrix for the seven risk factors. Principal component analysis was then performed using the correlation matrix, retaining only components with an eigenvalue ≥1. Factor loadings were then squared and summed to estimate communalities.
Step 3	The formula used for calculating **overall PAF** was as follows: $PAF=1-\left[\left(1-w^{*}{PAF}_{1}\right)\left(1-{w^{*}PAF}_{2}\right)\left(1-{w^{*}PAF}_{3}\right)\ldots \right]$ where weight (w) = 1-communality, allowing the adjusted PAFs to account for each risk factor's unique (non-shared) contribution.

PAFs were further stratified by sex, household income, migration background, and region. For each subgroup, prevalence and communalities were estimated separately, while RRs for each risk factor were the same as those applied in the primary analyses. All data were analysed using Stata statistical software, version 16·0 (StataCorp., College Station, Texas, United States of America).

### Role of the funding source

The funders had no involvement in study design, data collection, data analyses, data interpretation, or the writing of the report.

## Results

The overall Public Health Monitor survey response rates were 45–50% in 2012,^20^ 40% in 2016,^21^ 39% in 2020,^22^ and 32% in 2022.^23^ The final analytic samples retained 72·0–84·2% of respondents, yielding sample sizes of 302,422 (2012), 363,856 (2016), 409,278 (2020), and 314,647 (2022). Table 1 presents the sociodemographic characteristics for the four Public Health Monitor survey years. The country of origin of individuals with a migration background is provided in the Supplementary Materials (Figure S2 and Table S2). Across survey years, compared with included respondents, excluded respondents were generally older, more often female, more likely to have low educational attainment and lower household income, more frequently first-generation migrants, and widowed (Table S3). With regard to modifiable dementia risk factors, excluded respondents also showed a higher prevalence of obesity, physical inactivity, depression, and social isolation (Table S3).

**Table 1:** Sociodemographic characteristics among the study samples in the Netherlands in 2012, 2016, 2020 and 2022.

Sociodemographic characteristics	Year			
	2012 (n = 302,422)	2016 (n = 363,856)	2020 (n = 409,278)	2022 (n = 314,647)
Age, mean (SD)	55·8 (17·6)	59·5 (17·0)	57·7 (18·2)	59·0 (17·5)
Female, n (%)	161,634 (53·5)	193,101 (53·1)	215,054 (52·5)	165,451 (52·6)
Male, n (%)	140,788 (46·6)	170,755 (46·9)	194,224 (47·5)	149,196 (47·4)
Educational attainment, n (%)				
Low	124,539 (41·2)	136,705 (37·6)	130,898 (32·0)	96,484 (30·7)
Middle	89,081 (29·5)	114,236 (31·4)	133,397 (32·6)	98,814 (31·4)
High	88,802 (29·4)	112,915 (31·0)	144,983 (35·4)	119,349 (37·9)
Household income^a^^,^^b^ n (%)				
1st quantile (lowest)	28,067 (9·3)	27,367 (7·6)	36,500 (9·0)	26,875 (8·6)
2nd quantile	51,042 (17·0)	64,479 (17·8)	69,851 (17·2)	52,918 (17·0)
3rd quantile	63,445 (21·1)	78,790 (21·8)	86,169 (21·3)	66,828 (21·4)
4th quantile	74,903 (24·9)	90,954 (25·2)	99,992 (24·7)	78,340 (25·1)
5th quantile (highest)	83,175 (27·7)	100,075 (27·7)	113,058 (27·9)	87,054 (27·9)
Migration background, n (%)				
Dutch origin	261,147 (86·4)	316,301 (86·9)	350,093 (85·6)	271,898 (86·4)
First-generation migrant	22,604 (7·5)	26,012 (7·2)	33,683 (8·2)	24,132 (7·7)
Second-generation migrant	18,671 (6·2)	21,543 (5·9)	25,502 (6·2)	18,671 (5·9)
Marital status^a^, n (%)				
Married or cohabiting	219,131 (72·8)	262,364 (72·6)	291,558 (71·6)	192,491 (61·2)
Never married	36,480 (12·1)	38,567 (10·7)	55,834 (13·7)	67,436 (21·4)
Divorced	18,803 (6·3)	24,892 (6·9)	26,318 (6·5)	29,103 (9·3)
Widowed	26,595 (8·8)	35,773 (9·9)	33,713 (8·3)	25,615 (8·1)

Abbreviations: SD: standard deviation. Percentages may not sum to 100 due to rounding.

a Contains missing values.

b Quantiles are standardised from national income data in the Netherlands.

Weighted prevalences for each modifiable dementia risk factor, grouped by their life-course categories, are shown in Table 2. Over the past decade, weighted prevalences of low educational attainment, excessive alcohol consumption, and smoking decreased. In contrast, weighted prevalences of obesity, physical inactivity, depression, and social isolation increased.

**Table 2:** Weighted prevalences of modifiable dementia risk factors in the Netherlands in 2012, 2016, 2020, and 2022.

	2012	2016	2020	2022
**Early-life risk factors (<45 years)**				
Low educational attainment	18·3%	12·6%	11·0%	11·5%
**Mid-life risk factors (45–65 years)**				
Obesity	15·1%	16·5%	18·4%	18·9%
Physical inactivity	40·0%	38·5%	41·0%	44·5%
Excessive alcohol consumption	36·4%	31·5%	26·4%	26·5%
Smoking	22·6%	20·3%	16·1%	15·5%
Depression	5·2%	6·4%	5·8%	8·3%
**Late-life risk factors (>65 years)**				
Social isolation	44·2%	47·5%	48·8%	49·1%

Weighted prevalences refer to adjusted estimates intended to reflect the adult population in the Netherlands.

### Overall PAF trends

**Fig. 1:**
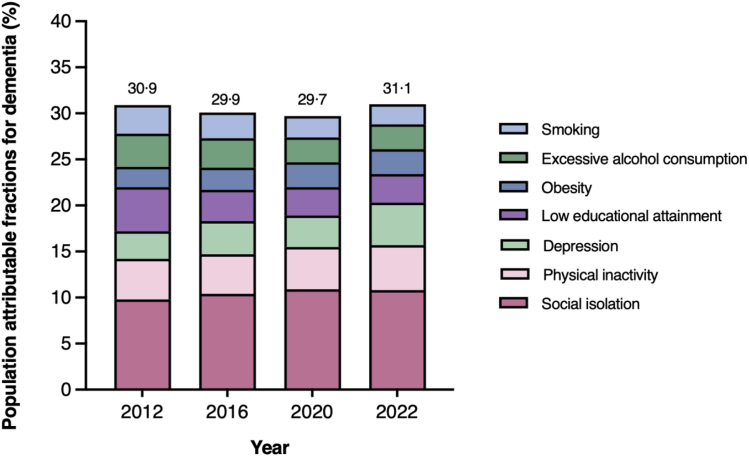
**Population attributable fractions of seven modifiable risk factors for dementia in the Netherlands in 2012, 2016, 2020 and 2022**.

In the Netherlands, the total PAFs for dementia from seven modifiable risk factors remained relatively stable over the decade, with estimates of 30·9% [16·8–43·4] in 2012, 29·9% [16·4–42·1] in 2016, 29·7% [16·5–41·4] in 2020, and 31·1% [17·7–43·2] in 2022 (Fig. 1). However, the contribution of individual dementia risk factors shifted over time. Between 2012 and 2022, individual PAFs for low educational attainment (4·8% to 3·1%), smoking (3·1% to 2·2%), and excessive alcohol consumption (3·6% to 2·7%) decreased. In comparison, PAFs for obesity (2·2% to 2·7%), physical inactivity (4·4% to 4·9%), depression (3·0% to 4·6%), and social isolation (9·8% to 10·8%) increased, with social isolation contributing the most to total PAFs throughout the decade. Further details on the PAF estimates and their corresponding communalities are provided in the Supplementary Materials (Tables S4–S7).

### Sex-specific PAFs

**Fig. 2:**
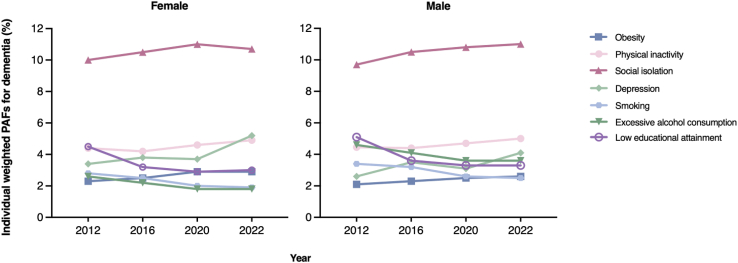
**Individual weighted population attributable fractions (PAFs) of seven modifiable risk factors for dementia, stratified by sex, in the Netherlands in 2012, 2016, 2020, and 2022**.

Overall, the sex-specific PAFs showed that males consistently had slightly higher PAFs than females across all years. In 2012, males had a PAF of 32·0% [17·7–44·7] compared with 30·0% [16·5–42·2] in females. This pattern persisted in 2016 (31·5% [17·8–43·7] vs 29·1% [16·1–41·0]), 2020 (30·5% [17·4–42·2] vs 29·0% [16·3–40·5]), and 2022 (32·1% [18·5–44·0] vs 30·4% [17·3–42·3]) (Table S8). While smoking and excessive alcohol consumption contributed more to male PAFs, patterns among females were more subtle, with a higher contribution from depression (Fig. 2).

### Household income-specific PAFs

**Fig. 3:**
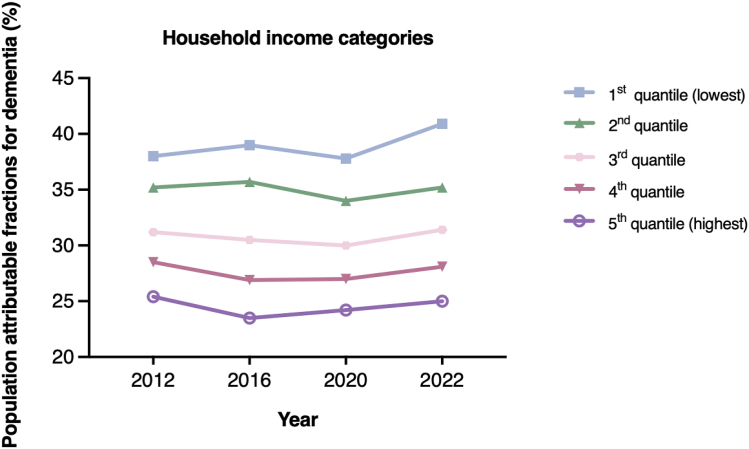
**Population attributable fractions of seven modifiable risk factors for dementia stratified by household income in the Netherlands in 2012, 2016, 2020 and 2022**.

Total PAFs for dementia showed a clear gradient across household income groups, with lower household income categories exhibiting higher PAF estimates over the years (Fig. 3). PAFs ranged from 38·0% [21·7–51·8] to 40·9% [24·2–54·6] in the lowest household income group and 23·5% [12·4–33·8] to 25·4% [13·5–36·2] in the highest (Tables S9–S12). Across increasing household income quantiles, individual PAFs for low educational attainment, smoking, social isolation, depression, and obesity generally declined (Figure S3). In contrast, the PAF for excessive alcohol consumption increased with income, while physical inactivity remained relatively stable across groups, particularly in 2020 and 2022.

### Migration background-specific PAFs

**Fig. 4:**
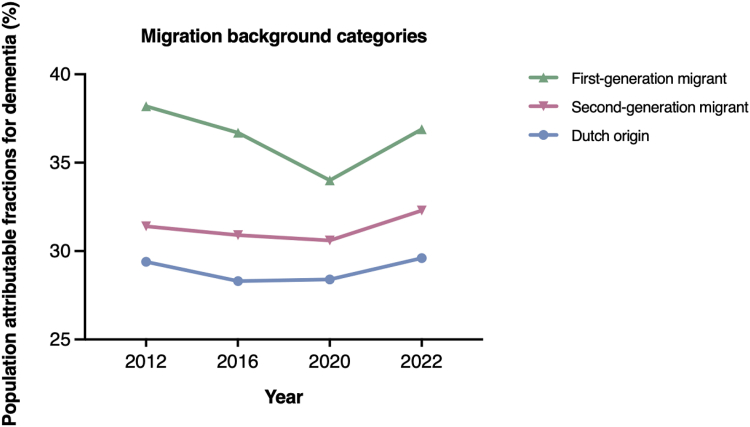
**Population attributable fractions of seven modifiable risk factors for dementia stratified by migration background in the Netherlands in 2012, 2016, 2020 and 2022**.

PAFs for dementia varied across migration backgrounds, with individuals of Dutch origin showing the lowest estimates (28·3% [15·3–40·2] to 29·6% [16·6–41·4]), followed by second-generation migrants (30·6% [17·6–42·3] to 32·3% [18·6–44·4]), and first-generation migrants displaying the highest values (34·0% [20·0–46·2] to 38·2% [22·3–51·8]) across the decade (Tables S13–S16). All groups showed declining trajectories up to 2020, with the steepest reduction among first-generation migrants, followed by an increase in 2022 across all groups (Fig. 4). First-generation migrants also had higher individual PAFs for depression and low educational attainment across the years, whereas Dutch origin and second-generation migrants showed more similar overall contributions (Figure S4).

### Region-specific PAFs

Region-specific PAFs fluctuated over time, with no region consistently showing the highest or lowest estimates across the decade (Figure S5). As for the total population, PAFs generally remained stable (Table S17). The regions with the highest and lowest estimates varied from year to year (Table S17). Details on individual weighted PAFs for dementia over the years for each region can be found in the Supplementary Materials (Figures S6–S30).

## Discussion

This cross-sectional, time series analysis provides estimates of PAFs for dementia in the Netherlands over a 10-year period and suggests that up to 31·1% of dementia cases may potentially be attributable to seven modifiable risk factors. Overall, PAFs remained relatively stable over time, though the contribution of individual modifiable risk factors varied. Males consistently exhibited slightly higher PAFs than females. A socioeconomic gradient was observed: individuals in the lowest household income group had the highest PAFs. Differences by migration background were also apparent. Individuals of Dutch origin and second-generation migrants had broadly comparable PAFs, with individuals of Dutch origin showing the lowest estimates, whereas first-generation migrants consistently demonstrated higher PAFs. Regional variation was likewise evident, indicating that PAFs for dementia were not uniform across the country. These findings highlight the potential population burden of modifiable dementia risk factors in the Netherlands and underscore that observed differences by sex, household income, migration background, and region may help inform tailored risk reduction approaches at both individual and population levels.

Between 2012 and 2022, overall dementia PAFs in the Netherlands remained relatively stable. Comparable stability has been reported in Australia, where estimates based on 15 risk factors were 47·2% in 2007–08 and 46·9% in 2022.^24^ In contrast, England observed a decline from 46·7% in 2004 to 36·8% in 2019, based on 12 risk factors.^25^ However, the latter study concluded before the onset of the COVID-19 pandemic, which may have altered the contributions of modifiable risk factors in more recent years.

Although the overall PAFs remained stable in the Netherlands, the contribution of individual modifiable risk factors shifted over time. Social isolation consistently represented the single largest contributor to the overall PAF across years and across all sociodemographic subgroups. It should be noted that the prevalence of social isolation in the current study (44⋅2% in 2012 to 49⋅1% in 2022) is higher than reported in previous research. For example, a meta-analysis of 41 studies (n = 183,250) reported a global prevalence of 24⋅0%.^26^ This discrepancy may be explained by heterogeneity in measurement methods. Prior studies often use a variety of instruments to measure social isolation, such as the Lubben Social Network Scale or self-developed questionnaires,^26^ whereas the current study employed the De Jong Gierveld Loneliness Scale (score of ≥3), which captures loneliness, a subjective feeling of social isolation.^27^

PAFs for low educational attainment, excessive alcohol consumption, and smoking decreased. The decline in low educational attainment may potentially reflect generational transitions linked to policy reforms, such as the extension of compulsory education (Compulsory Education Act) from age 5–12 to 5–16 in 1969 and the introduction of a basic qualification requirement in 2007 aimed at reducing early school leaving. Reductions in PAFs for excessive alcohol consumption and smoking may potentially correspond to strengthened public health regulations, such as increasing excise duties on cigarettes and alcohol over the past two decades.

By contrast, PAFs for obesity and physical inactivity increased over the decade, possibly reflecting broader changes in the built and food environment^28^ that promote sedentary lifestyles. Rising PAFs for depression and social isolation may reflect higher levels of urbanisation, lower socioeconomic neighbourhood conditions (including social safety),^29^ and the impact of the COVID-19 pandemic.^30^ However, these trends may reflect multiple contributing factors beyond those discussed, including behavioural and economic changes; thus, these findings warrant cautious interpretation.

PAF estimates for males and females were broadly comparable, consistent with findings based on Chinese and Canadian samples, presenting minimal sex-specific differences in PAFs.^31^^,^^32^ Nonetheless, males showed higher individual PAFs for substance use-related factors (excessive alcohol consumption and smoking). This pattern may be related to male-dominated occupational settings and masculine gender norms that encourage risk-taking behaviours.^33^^,^^34^ In contrast, females had a higher individual PAF for depression, which may be linked to hormonal fluctuations (e.g., menstrual cycle and menopause),^35^ gender-role expectations, and broader sociocultural factors, including discrimination and inequality.^36^

Higher PAFs for dementia were observed among lower household income groups, aligning with prior research suggesting that lower socioeconomic position is associated with a higher risk.^37^ Although social isolation contributed the most to the overall PAFs, its relative impact did not vary substantially across income categories. In contrast, lower household incomes exhibited higher individual PAFs for low educational attainment and depression, consistent with findings from Australia.^24^ These differences could reflect long-term socioeconomic disadvantage, in which limited educational opportunities (shaped by family background) constrain income potential^38^ and contribute to chronic stress arising from financial difficulties, housing insecurity, and limited access to healthcare.^37^ Meanwhile, individual PAFs for excessive alcohol consumption increased with household income. Individuals with a higher socioeconomic position have been reported to consume more alcohol than those with a lower socioeconomic position, which may be related to higher disposable income and social contexts that facilitate alcohol consumption.^39^

First-generation migrants exhibited the highest PAFs for dementia, followed by second-generation migrants and individuals of Dutch origin, with only modest differences between the latter two groups. No previous studies have specifically examined dementia PAFs by migration background. However, findings from the Healthy Life in an Urban Setting (HELIUS) study in the Netherlands show that, compared with individuals of Dutch origin, those with a migration background had a higher dementia risk across three composite risk scores.^10^ In the current study, depression and low educational attainment contributed more strongly to overall dementia PAFs among first-generation migrants than among second-generation migrants and individuals of Dutch origin. For depression, this may conceivably relate to stressors experienced before and after migration, including cultural transitions, limited social networks, financial strain,^40^ and discrimination. For low educational attainment, this may reflect host-country language proficiency and (quality of) educational trajectories before migration, including differences in educational systems across countries of origin.^41^

By contrast, second-generation migrants and individuals of Dutch origin showed similar overall PAFs and comparable risk–factor profiles, highlighting the potential influence of integration and Dutch language proficiency. Nonetheless, these findings underscore the need for further research, as substantial knowledge gaps remain in dementia research among migrant populations.

Within the Netherlands, regional variation in PAFs for dementia was observed. Although a rural-urban divide exists, with areas in the densely populated ‘Randstad’ (Amsterdam, Haaglanden, Rotterdam-Rijnmond and Utrecht) considered the country's economic, cultural and political centre, PAFs for dementia did not necessarily reflect this distinction. The region of Limburg (Limburg-Noord and Zuid-Limburg), for example, consistently had higher PAFs for dementia than other regions, which aligns with various health reports indicating that residents of this region are, on average, less healthy and live, on average, 1 year shorter than other individuals in the Netherlands.^42^ These findings suggest that, even within a single country, disparities in dementia risk exist, underscoring the important role and responsibility of national and local governments (municipalities) in shaping policies that support healthy ageing.

The current study has several strengths. It quantifies the potential for dementia risk reduction in the Netherlands over a ten-year period, as well as across sociodemographic subgroups (i.e., sex, household income, and migration background) and regions. The study draws on large samples, averaging approximately 350,000 individuals per year, with survey-specific weights applied to enhance representativeness of the Dutch adult population.

Nonetheless, several limitations should be acknowledged. First, the study relies on self-reported data, which are subject to under-reporting bias, particularly for socially sensitive risk factors such as depression and excessive alcohol consumption, and may have resulted in conservative PAFs. Second, PAFs calculated using Levin's formula assume that the RR estimates are causal relationships and have no residual confounding.^43^ However, this assumption is unlikely to be fully met in observational data, particularly for complex risk factors such as depression^44^ and social isolation, where reverse causation is possible. The estimated PAFs should, therefore, be interpreted cautiously. Third, the RRs used to estimate PAFs were derived from robust meta-analyses and based on worldwide data. As robust subgroup-specific RR estimates are absent (including for the Dutch population), differences in PAFs mainly reflect variations in risk factor prevalences and communalities. Future longitudinal studies in the Netherlands, including within sociodemographic subgroups, are needed to refine these estimates. Fourth, due to data unavailability, PAF estimates were based on only seven modifiable risk factors, whereas the literature has identified more potentially modifiable dementia risk factors. Consequently, the estimated PAFs are likely conservative and do not capture the full potential burden of modifiable dementia risk factors in the Netherlands. Finally, while survey-specific weights were applied, restricting the analysis to respondents with complete data on all seven risk factors may have introduced selection bias. Consequently, the analytic samples may not fully represent the Dutch adult population, which may limit the generalisability of the findings and lead to the underestimation of some PAFs.

This study provides comprehensive estimates of potentially modifiable dementia risk in the Netherlands over a 10-year period attributable to seven common risk factors. Although declines were observed in several modifiable risk factors, namely low educational attainment, smoking, and excessive alcohol consumption, increases in social isolation, depression, obesity and physical inactivity counterbalanced these trends, resulting in stable PAFs for dementia over time. PAFs varied by sex, household income, migration background, and region, underscoring the need for well-designed, targeted public health interventions. Notably, such interventions must be implemented with careful consideration to avoid exacerbating existing social and health inequalities. From a feasibility perspective, risk reduction strategies that address the environments in which people live are likely to have the greatest impact, as these population-level approaches can reach large segments of the population while limiting reliance on substantial resources or individual behaviour change.^45^

## Contributors

TLVB led the study's conceptualisation and design, conducted the data analysis, interpreted the findings, drafted, reviewed, and edited the manuscript. KD was involved in the conceptualisation and design of the study, contributed to the interpretation of the findings, and reviewed and edited the manuscript. NJ was involved in the conceptualisation and design of the study, assisted with the data analysis, interpreted the findings, and drafted, reviewed, and edited the manuscript. LAD assisted with the data analysis, contributed to the interpretation of the findings, and reviewed and edited the manuscript. SK contributed to the interpretation of the findings and reviewed and edited the manuscript. Due to restrictions on accessing the Statistics Netherlands remote environment, only TLVB, KD, NJ, and SK had access to view the data. TLVB was the only author authorised to analyse the data and conduct all data analyses. KD, NJ, and SK have verified the underlying data. All authors have read and approved the final version of the manuscript.

## Data sharing statement

The dataset was provided by Statistics Netherlands (CBS) and the Dutch Public Health Services. Requests to access these datasets should be directed to Statistics Netherlands. Results are based on calculations by researchers from Maastricht University in project number 9716 using non-public microdata from Statistics Netherlands.

## Editor note

The Lancet Group takes a neutral position with respect to territorial claims in published maps and institutional affiliations.

## Declaration of interests

TLVB has received a travel grant from Alzheimer Nederland.

KD has received grants from the Netherlands Organisation for Health Research and Development (ZonMw) and the Alzheimer's Association. KD is also a Member of the Advisory Board for the National Dementia Strategy 2021–2030 (Dutch Ministry of Health, Welfare and Sport, 2021–2025), a Member of the Expert Advisory Panel of Alzheimer Europe, and a Member of the Evidence Review Team, Brain Health Unit, World Health Organisation (WHO).

NJ has received grants from ZonMw and is a Member of the Evidence Review Team, Brain Health Unit, WHO.

LAD has received a travel grant from Alzheimer Nederland and is a Member of the Evidence Review Team, Brain Health Unit, WHO.

SK has received grants from ZonMw, Maastricht University, and the Netherlands Scientific Organisation. SK is also a Member of the Scientific Advisory Board “Plan Dementia Prevention,” Chair of the Early, Timely INTERventions in DEMentia (INTERDEM) Preventon Task Force, a Member of the Expert Advisory Panel of Alzheimer Europe, Expert Consultant to the WHO Brain Health Unit, Chair of the WHO Guidelines Development Group on Risk Reduction of Cognitive Decline and Dementia, and an Advisor to the Dutch Health Council (Gezondheidsraad).

## References

[1] den Broeder L., Hilderink H., Polder J. (2024). https://www.rivm.nl/bibliotheek/rapporten/2024-0203.pdf.

[2] Alzheimer Nederland (2025). Feiten en cijfers over dementie. https://www.alzheimer-nederland.nl/dementie/feiten-en-cijfers-over-dementie.

[3] Ministry of Health, Welfare and Sport (2020). https://www.alzint.org/u/Netherlands-NationalDementiaStrategy2021-2030.pdf.

[4] Francke A., van der Heide I., de Bruin S. (2018). https://www.nivel.nl/nl/publicatie/een-samenhangend-beeld-van-dementie-en-dementiezorg-kerncijfers-behoeften-aanbod-en.

[5] Livingston G., Huntley J., Liu K.Y. (2024). Dementia prevention, intervention, and care: 2024 report of the Lancet standing Commission. Lancet.

[6] Stephan B.C.M., Cochrane L., Kafadar A.H. (2024). Population attributable fractions of modifiable risk factors for dementia: a systematic review and meta-analysis. Lancet Healthy Longev.

[7] Wang A.Y., Hu H.Y., Ou Y.N. (2023). Socioeconomic status and risks of cognitive impairment and dementia: a systematic review and meta-analysis of 39 prospective studies. J Prev Alzheimers Dis.

[8] Gong J., Harris K., Lipnicki D.M. (2023). Sex differences in dementia risk and risk factors: Individual-participant data analysis using 21 cohorts across six continents from the COSMIC consortium. Alzheimer's Dement.

[9] Riedel B.C., Thompson P.M., Brinton R.D. (2016). Age, APOE and sex: triad of risk of Alzheimer's disease. J Steroid Biochem Mol Biol.

[10] Lindhout J., van der Endt A.R., Hoevenaar-Blom M.P. (2025). Midlife dementia risk scores in a multi-ethnic population in the Netherlands: the HELIUS study. J Public Health.

[11] Huijsmans T. (2023). Place resentment in ‘the places that don't matter’: explaining the geographic divide in populist and anti-immigration attitudes. Acta Politic.

[12] Rijksinstituut voor Volksgezondheid en Milieu Gezondheidsmonitor Volwassenen en Ouderen. https://www.monitorgezondheid.nl/gezondheidsmonitor-volwassenen-en-ouderen.

[13] Weggemans R.M., Backx F.J.G., Borghouts L. (2018). The 2017 Dutch Physical Activity Guidelines. Int J Behav Nutr Phys Act.

[14] Kessler R.C., Barker P.R., Colpe L.J. (2003). Screening for serious mental illness in the general population. Arch Gen Psychiatry.

[15] De Jong-Gierveld J., Van Tilburg T. (1999). https://research.vu.nl/ws/portalfiles/portal/1092113/1999%20dJG%20vT%20Loneliness%20manual.pdf.

[16] Buelens B., Meijers R., Tennekes M. Weging Gezondheidsmonitor 2012. Hague: Statistics Netherlands. https://www.cbs.nl/en-gb/our-services/customised-services-microdata/microdata-conducting-your-own-research.

[17] Buelens B (2017). Weging Gezondheidsmonitor 2016. The Hague: Statistics Netherlands. https://www.cbs.nl/en-gb/our-services/customised-services-microdata/microdata-conducting-your-own-research.

[18] Kierkels A. (2021). Weegrapport GM 2020. The Hague: Statistics Netherlands. https://www.cbs.nl/en-gb/our-services/customised-services-microdata/microdata-conducting-your-own-research.

[19] Theulings A (2023). Weging Gezondheidsmonitor Volwassenen en Ouderen 2022. The Hague: Statistics Netherlands. https://www.cbs.nl/en-gb/our-services/customised-services-microdata/microdata-conducting-your-own-research.

[20] Statistics Netherlands Gezondheidsmonitor. https://www.cbs.nl/nl-nl/onze-diensten/methoden/onderzoeksomschrijvingen/korte-onderzoeksomschrijvingen/gezondheidsmonitor.

[21] Statistics Netherlands Gezondheidsmonitor 2016. https://www.cbs.nl/nl-nl/onze-diensten/methoden/onderzoeksomschrijvingen/korte-onderzoeksomschrijvingen/gezondheidsmonitor-2016.

[22] Statistics Netherlands Gezondheidsmonitor Volwassenen en Ouderen 2020. https://www.cbs.nl/nl-nl/onze-diensten/methoden/onderzoeksomschrijvingen/korte-onderzoeksomschrijvingen/gezondheidsmonitor-volwassenen-en-ouderen-2020.

[23] Statistics Netherlands Gezondheidsmonitor Volwassenen en Ouderen 2022. https://www.cbs.nl/nl-nl/onze-diensten/methoden/onderzoeksomschrijvingen/korte-onderzoeksomschrijvingen/gezondheidsmonitor-volwassenen-en-ouderen-2022.

[24] Welberry H.J., Jorm L.R., Kiely K.M., Huque H., Peters R., Anstey K.J. (2025). Sex and socioeconomic differences in 15-year prevalence trends for modifiable dementia risk factors in Australia: a cross-sectional, time series analysis. Lancet Healthy Longev.

[25] Chen S., Underwood B.R., Cardinal R.N. (2024). Temporal trends in population attributable fractions of modifiable risk factors for dementia: a time-series study of the English Longitudinal Study of Ageing (2004–2019). BMC Med.

[26] Teo R.H., Cheng W.H., Cheng L.J., Lau Y., Lau S.T. (2023). Global prevalence of social isolation among community-dwelling older adults: a systematic review and meta-analysis. Arch Gerontol Geriatr.

[27] Danvers A.F., Efinger L.D., Mehl M.R. (2023). Loneliness and time alone in everyday life: a descriptive-exploratory study of subjective and objective social isolation. J Res Pers.

[28] Vandevijvere S., De Pauw R., Djojosoeparto S. (2023). Upstream determinants of overweight and obesity in Europe. Curr Obes Rep.

[29] Generaal E., Hoogendijk E.O., Stam M. (2019). Neighbourhood characteristics and prevalence and severity of depression: pooled analysis of eight Dutch cohort studies. Br J Psychiatry.

[30] van Tilburg T.G., Steinmetz S., Stolte E., van der Roest H., de Vries D.H. (2020). Loneliness and mental health during the COVID-19 pandemic: a study among Dutch older adults. J Gerontol B Pyschol Sci Soc Sci.

[31] Son S., Speechley M., Zou G.Y. (2024). Potentially modifiable dementia risk factors in Canada: an analysis of Canadian longitudinal Study on aging with a multi-country comparison. J Prev Alzheimers Dis.

[32] Chen S., Chen X., Hou X., Fang H., Liu G.G., Yan L.L. (2024). Temporal trends and disparities of population attributable fractions of modifiable risk factors for dementia in China: a time-series study of the China health and retirement longitudinal study (2011–2018). Lancet Reg Health West Pac.

[33] Mahalik J.R., Burns S.M., Syzdek M. (2007). Masculinity and perceived normative health behaviors as predictors of men's health behaviors. Soc Sci Med.

[34] Hulls P.M., Richmond R.C., Martin R.M., de Vocht F. (2020). A systematic review protocol examining workplace interventions that aim to improve employee health and wellbeing in male-dominated industries. Syst Rev.

[35] Kundakovic M., Rocks D. (2022). Sex hormone fluctuation and increased female risk for depression and anxiety disorders: from clinical evidence to molecular mechanisms. Front Neuroendocrinol.

[36] Baños R.M., Miragall M. (2024). Gender matters: a critical piece in mental health. Span J Psychol.

[37] Bodryzlova Y., Kim A., Michaud X., André C., Bélanger E., Moullec G. (2023). Social class and the risk of dementia: a systematic review and meta-analysis of the prospective longitudinal studies. Scand J Public Health.

[38] OECD (2024).

[39] Collins S.E. (2016). Associations between socioeconomic factors and alcohol outcomes. Alcohol Res.

[40] Kovaleva M., Jones A., Maxwell C.A. (2021). Immigrants and dementia: literature update. Geriatr Nurs.

[41] Bonin H. (2017). https://docs.iza.org/report_pdfs/iza_report_75.pdf.

[42] Jansen M., Kuppens E. (2015). https://www.ggdzl.nl/fileadmin/files/ggdzl/Documenten/Op-zoek-naar-de-Limburg-factor.pdf.

[43] Ferguson J., Alvarez A., Mulligan M., Judge C., O'Donnell M. (2024). Bias assessment and correction for Levin's population attributable fraction in the presence of confounding. Eur J Epidemiol.

[44] Singh-Manoux A., Dugravot A., Fournier A. (2017). Trajectories of depressive symptoms before diagnosis of dementia: a 28-Year Follow-up study. JAMA Psychiatry.

[45] Frisoni G.B., Aho E., Brayne C. (2025). Alzheimer's disease outlook: controversies and future directions. Lancet.

