# Supplementary material for: Population attributable fractions of modifiable dementia risk factors in the Netherlands: a cross-sectional, time series analysis
Source: eClinicalMedicine. 2026 Jun 16;96:104018. doi: 10.1016/j.eclinm.2026.104018 (PMC13284433; doi:10.1016/j.eclinm.2026.104018)
Supplement: Supplementary Material [file mmc1.pdf]

Supplementary Materials

Population attributable fractions of modifiable dementia risk factors in the Netherlands: a cross-sectional, time series analysis

Ms Tessa Lin van Baal MSc, Dr Niels Janssen PhD, Dr Lukas Andreas Duffner PhD, Prof Sebastian Köhler PhD, Dr Kay Deckers PhD

Table of contents

Table S1. Household income categories based on national income data from the previous year. 4

Figure S1. Map of the Netherlands showing the geographical distribution of municipal public health service (GGD) regions. 4

Figure S2. Classification of the country of origin.. 5

Table S2. Country of origin by migration background. 6

Table S3. Sociodemographic characteristics of the included and excluded analytical samples for the years 2012, 2016, 2020, and 2022. 7

Table S4. Population attributable fractions for seven modifiable risk factors for dementia in the Netherlands in 2012. 9

Table S5. Population attributable fractions for seven modifiable risk factors for dementia in the Netherlands in 2016. 10

Table S6. Population attributable fractions for seven modifiable risk factors for dementia in the Netherlands in 2020. 11

Table S7. Population attributable fractions for seven modifiable risk factors for dementia in the Netherlands in 2022. 12

Table S8. Total and year-specific population attributable fractions for modifiable dementia risk factors in the Netherlands, stratified by sex. 13

Table S9. Total population attributable fractions for modifiable dementia risk factors in the Netherlands in 2012, stratified by household income. 15

Table S10. Total population attributable fractions for modifiable dementia risk factors in the Netherlands in 2016, stratified by household income. 16

Table S11. Total population attributable fractions for modifiable dementia risk factors in the Netherlands in 2020, stratified by household income. 17

Table S12. Total population attributable fractions for modifiable dementia risk factors in the Netherlands in 2022, stratified by household income. 18

Figure S4: Weighted population attributable fractions for individual modifiable dementia risk factors, stratified by migration background. 19

Table S13. Total and population attributable fractions for modifiable dementia risk factors in the Netherlands in 2012, stratified by migration background. 20

Table S14. Total and population attributable fractions for modifiable dementia risk factors in the Netherlands in 2016, stratified by migration background. 20

Table S15. Total and population attributable fractions for modifiable dementia risk factors in the Netherlands in 2020, stratified by migration background. 21

Table S16. Total and population attributable fractions for modifiable dementia risk factors in the Netherlands in 2022, stratified by migration background. 21

Table S17. Total and year-specific population attributable fractions for modifiable dementia risk factors in the Netherlands, stratified by region. 22

Figure S5: Choropleth maps of population attributable fractions for seven modifiable dementia risk factors across 25 municipal public health service (GGD) regions of the Netherlands, 2012-2022 23

Figure S6. Individual weighted population attributable fractions of seven modifiable risk factors for dementia in municipal public health service (GGD) Groningen in 2012, 2016, 2020, and 2022.. 24

Figure S7. Individual weighted population attributable fractions of seven modifiable risk factors for dementia in municipal public health service (GGD) Drenthe in 2012, 2016, 2020, and 2022. 24

Figure S8. Individual weighted population attributable fractions of seven modifiable risk factors for dementia in municipal public health service (GGD) IJsselland in 2012, 2016, 2020, and 2022. 25

Figure S9. Individual weighted population attributable fractions of seven modifiable risk factors for dementia in municipal public health service (GGD) Twente in 2012, 2016, 2020, and 2022.. 25

Figure S10. Individual weighted population attributable fractions of seven modifiable risk factors for dementia in municipal public health service (GGD) Noord- en Oost-Gelderland in 2012, 2016, 2020, and 2022. 26

Figure S11. Individual weighted population attributable fractions of seven modifiable risk factors for dementia in Veiligheids- en Gezondheidsregio Gelderland-Midden in 2012, 2016, 2020, and 2022. 26

Figure S12. Individual weighted population attributable fractions of seven modifiable risk factors for dementia in municipal public health service (GGD) Gelderland-Zuid in 2012, 2016, 2020, and 2022. 27

Figure S13. Individual weighted population attributable fractions of seven modifiable risk factors for dementia in municipal public health service (GGD) Flevoland in 2012, 2016, 2020, and 2022. 27

Figure S14. Individual weighted population attributable fractions of seven modifiable risk factors for dementia in municipal public health service (GGD) Regio Utrecht in 2012, 2016, 2020, and 2022.. 28

Figure S15. Individual weighted population attributable fractions of seven modifiable risk factors for dementia in municipal public health service (GGD) Hollands-Noorden in 2012, 2016, 2020, and 2022.. 28

Figure S16. Individual weighted population attributable fractions of seven modifiable risk factors for dementia in municipal public health service (GGD) Kennemerland in 2012, 2016, 2020, and 2022. 29

Figure S17. Individual weighted population attributable fractions of seven modifiable risk factors for dementia in municipal public health service (GGD) Amsterdam in 2012, 2016, 2020, and 2022. 29

Figure S18. Individual weighted population attributable fractions of seven modifiable risk factors for dementia in municipal public health service (GGD) Gooi en Vechtstreek in 2012, 2016, 2020, and 2022.. 30

Figure S19. Individual weighted population attributable fractions of seven modifiable risk factors for dementia in municipal public health service (GGD) Hollands-Midden in 2012, 2016, 2020, and 2022..... 30

Figure S20. Individual weighted population attributable fractions of seven modifiable risk factors for dementia in municipal public health service (GGD) Rotterdam-Rijnmond in 2012, 2016, 2020, and 2022. 31

Figure S21. Individual weighted population attributable fractions of seven modifiable risk factors for dementia in Dienst Gezondheid & Jeugd Zuid-Holland Zuid in 2012, 2016, 2020, and 2022.. 31

Figure S22. Individual weighted population attributable fractions of seven modifiable risk factors for dementia in municipal public health service (GGD) Zeeland in 2012, 2016, 2020, and 2022. 32

Figure S23. Individual weighted population attributable fractions of seven modifiable risk factors for dementia in municipal public health service (GGD) West-Brabant in 2012, 2016, 2020, and 2022.. 32

Figure S24. Individual weighted population attributable fractions of seven modifiable risk factors for dementia in municipal public health service (GGD) Hart voor Brabant in 2012, 2016, 2020, and 2022.. . 33

Figure S25. Individual weighted population attributable fractions of seven modifiable risk factors for dementia in municipal public health service (GGD) Brabant-Zuidoost in 2012, 2016, 2020, and 2022.. ... 33

Figure S26. Individual weighted population attributable fractions of seven modifiable risk factors for dementia in municipal public health service (GGD) Limburg-Noord in 2012, 2016, 2020, and 2022. 34

Figure S27. Individual weighted population attributable fractions of seven modifiable risk factors for dementia in municipal public health service (GGD) Zuid-Limburg in 2012, 2016, 2020, and 2022.. 34

Figure S28. Individual weighted population attributable fractions of seven modifiable risk factors for dementia in municipal public health service (GGD) Haaglanden in 2012, 2016, 2020, and 2022.. 35

Figure S29. Individual weighted population attributable fractions of seven modifiable risk factors for dementia in municipal public health service (GGD) Fryslân in 2012, 2016, 2020, and 2022. 35

Figure S30. Individual weighted population attributable fractions of seven modifiable risk factors for dementia in municipal public health service (GGD) Zaanstreek/Waterland in 2012, 2016, 2020, and 2022 36

Table S1. Household income categories based on national income data from the previous year.

	2012*	2016	2020	2022
**1st quantile (lowest)**	0-20% (max €15·200)	0-20% (max €16·100)	0-20% (max €18·200)	0-20% (max €19·600)
**2nd quantile**	20-40% (max €19·400)	20-40% (max €21·300)	20-40% (max €24·200)	20-40% (max €26·300)
**3rd quantile**	40-60% (max €24·200)	40-60% (max €27·200)	40-60% (max €30·800)	40-60% (max €33·500)
**4th quantile**	60-80% (max €31·000)	60-80% (max €35·100)	60-80% (max €39·500)	60-80% (max €43·000)
**5th quantile (highest)**	80-100% (>€31·000)	80-100% (>€35·100)	80-100% (>€39·500)	80-100% (>€43·000)

* Based on 2010 national income data

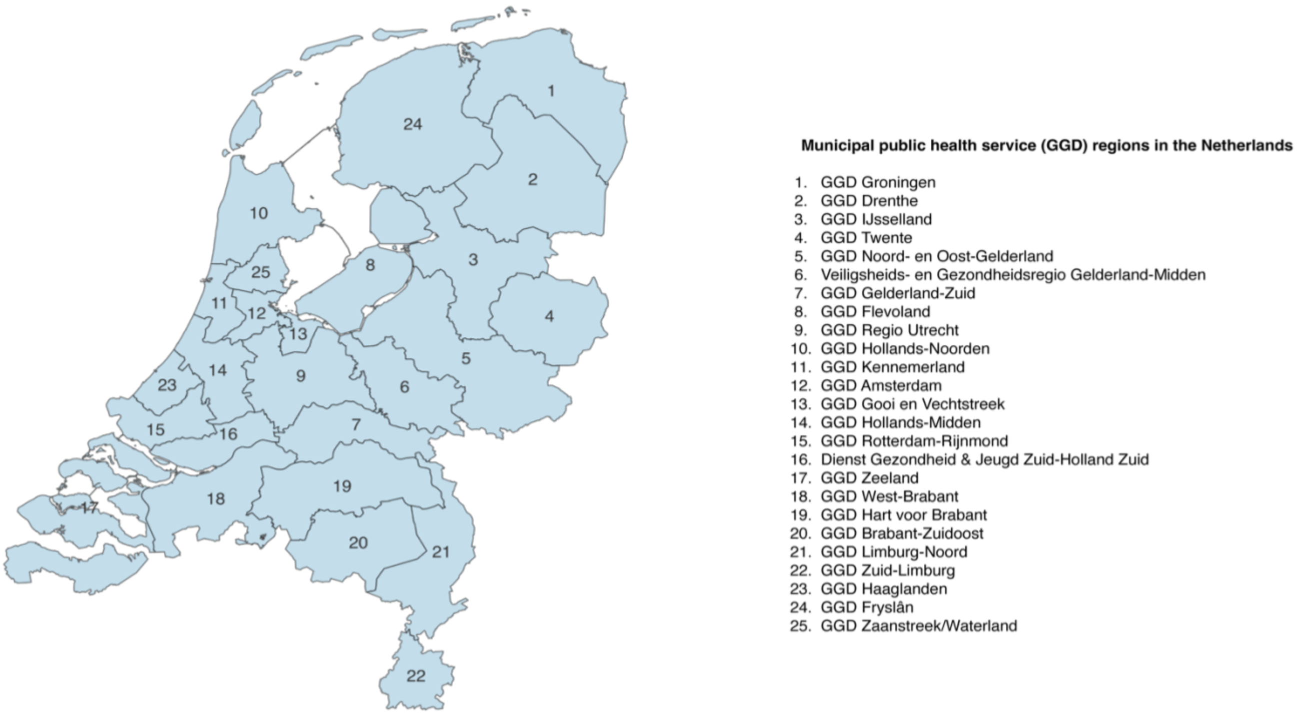

Figure S1. Map of the Netherlands showing the geographical distribution of municipal public health service (GGD) regions.

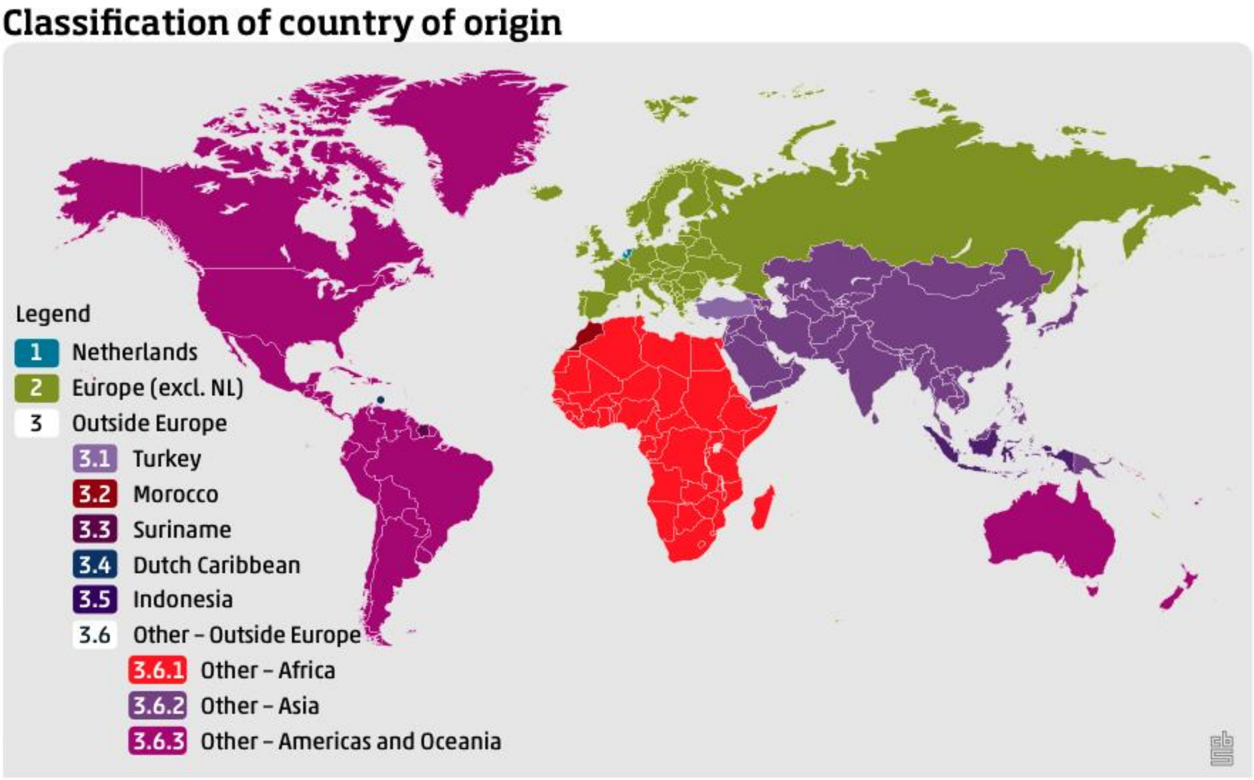

Figure S2. Classification of the country of origin. ***Abbreviations: excl: excluding; NL: Netherlands.***

Table S2. Country of origin by migration background.

	First-generation migrant				Second-generation migrant			
	**2012[a]**	**2016[b]**	**2020[c]**	**2022[d]**	**2012[e]**	**2016[f]**	**2020[g]**	**2022[h]**
**Country of origin, n (%)**								
Europe (excluding the Netherlands)	7,240 (32·9)	9,236 (35·6)	12,581 (37·4)	9,099 (37·8)	11,247 (60·3)	13,311 (61·8)	13,676 (53·7)	9,364 (50·4)
Turkey	1,784 (7·9)	1,461 (5·6)	1,693 (5·0)	1,192 (4·9)	703 (3·8)	641 (3·0)	961 (3·8)	708 (3·8)
Morocco	1,375 (6·1)	1,068 (4·1)	1,093 (3·3)	743 (3·1)	356 (1·9)	348 (1·6)	529 (2·1)	374 (2·0)
Suriname	2,036 (9·0)	2,376 (9·2)	2,592 (7·7)	1,741 (7·2)	786 (4·2)	753 (3·5)	1,241 (4·9)	719 (3·9)
Dutch Caribbean	984 (4·4)	990 (3·8)	1,215 (3·6)	950 (3·9)	304 (1·6)	297 (1·4)	462 (1·8)	426 (2·3)
Indonesia	3,671 (16·3)	4,493 (17·3)	3,978 (11·8)	2,775 (11·5)	4,134 (22·2)	4,977 (23·1)	6,269 (24·6)	5,375 (28·9)
Other Africa	1,293 (5·7)	1,428 (5·5)	2,064 (6·1)	1,444 (6·0)	287 (1·5)	260 (1·2)	539 (2·1)	343 (1·8)
Other Asia	2,808 (12·5)	3,393 (13·1)	5,929 (17·6)	4,172 (17·3)	420 (2·3)	451 (2·1)	941 (3·7)	659 (3·5)
Other Americas and Oceania	1,189 (5·3)	1,512 (5·8)	2,484 (7·4)	1,990 (8·3)	427 (2·3)	492 (2·3)	861 (3·4)	630 (3.4)

Missing data (country of origin unavailable) 44[a]; 55[b]; 54[c]; 26[d]; <10[e]; 13[f]; 23[g]; 19[h]

Table S3. Sociodemographic characteristics of the included and excluded analytical samples for the years 2012, 2016, 2020, and 2022.

	Year							
	2012		2016		2020		2022	
	Included *(n=302,422)*	Excluded *(n=84,773)*	Included *(n=363,856)*	Excluded *(n=93,297)*	Included *(n=409,278)*	Excluded *(n=130,624)*	Included *(n=314,647)*	Excluded *(n=49,910)*
**Sociodemographic characteristics**								
Age, mean (SD)	55.8 (17.6)	63.1 (18.2)	59.5 (17.0)	64.5 (18.0)	57.7 (18.2)	61.0 (19.4)	59.0 (17.5)	62.3 (18.9)
Female, n (%)	161,634 (53.5)	49,655 (58.6)	193,101 (53.1)	54,018 (57.9)	215,054 (52.5)	71,342 (54.6)	165,451 (52.6)	28,172 (56.5)
Educational attainment, n (%)								
Low	124,539 (41.2)	43,678 (61.0)	136,705 (37.6)	36,349 (58.8)	130,898 (32.0)	41,372 (42.1)	96,484 (30.7)	22,184 (47.7)
Middle	89,081 (29.5)	16,782 (23.5)	114,236 (31.4)	15,863 (25.6)	133,397 (32.6)	28,555 (29.1)	98,814 (31.4)	13,927 (29.9)
High	88,802 (29.4)	11,116 (15.5)	112,915 (31.0)	9,663 (15.6)	144,983 (35.4)	28,287 (28.8)	119,349 (37.9)	10,413 (22.4)
Household income, n (%)								
1st quantile (lowest)	28,067 (9.3)	11,005 (13.1)	27,367 (7.6)	10,629 (11.5)	36,500 (9.0)	15,086 (11.7)	26,875 (8.6)	7,261 (14.8)
2nd quantile	51,042 (17.0)	23,395 (27.8)	64,479 (17.8)	25,996 (28.1)	69,851 (17.2)	28,771 (22.2)	52,918 (17.0)	12,327 (25.1)
3rd quantile	63,445 (21.1)	18,709 (22.2)	78,790 (21.8)	21,650 (23.4)	86,169 (21.3)	27,115 (21.0)	66,828 (21.4)	10,875 (22.1)
4th quantile	74,903 (24.9)	16,472 (19.6)	90,954 (25.2)	18,211 (19.7)	99,992 (24.7)	27,959 (21.6)	78,340 (25.1)	9,770 (19.9)
5th quantile (highest)	83,175 (27.7)	14,630 (17.4)	100,075 (27.7)	16,125 (17.4)	113,058 (27.9)	30,464 (23.5)	87,054 (27.9)	8,940 (18.2)
Migration background, n (%)								
Dutch origin	261,147 (86.4)	71,124 (83.9)	316,301 (86.9)	78,240 (83.9)	350,093 (85.6)	109,631 (83.9)	271,898 (86.4)	40,302 (80.8)
First-generation migrant	22,604 (7.5)	8,379 (9.9)	26,012 (7.2)	9,358 (10.0)	33,683 (8.2)	12,829 (9.8)	24,132 (7.7)	6,519 (13.1)
Second-generation migrant	18,671 (6.2)	5,227 (6.2)	21,543 (5.9)	5,699 (6.1)	25,502 (6.2)	8,164 (6.3)	18,617 (5.9)	3,089 (6.2)
Marital status, n (%)								
Married or cohabiting	219,131 (72.8)	43,822 (63.2)	262,364 (72.6)	53,900 (63.9)	291,558 (71.6)	80,234 (66.4)	192,491 (61.2)	27,949 (56.0)
Never married	36,480 (12.1)	6,464 (9.3)	38,567 (10.7)	8,033 (9.5)	55,834 (13.7)	15,587 (12.9)	67,436 (21.4)	10,139 (20.3)
Divorced	18,803 (6.3)	4,621 (6.7)	24,892 (6.9)	5,970 (7.1)	26,318 (6.5)	8,270 (6.8)	29,103 (9.3)	4,801 (9.6)
Widowed	26,595 (8.8)	14,474 (20.9)	35,773 (9.9)	16,462 (19.5)	33,713 (8.3)	16,735 (13.9)	25,615 (8.1)	7,010 (14.1)
Dementia risk factors, n (%)								
Obesity	40,044 (13.2)	10.595 (15.3)	53,914 (14.8)	12,023 (16.7)	62,595 (15.3)	17,693 (15.3)	50,082 (15.9)	5,879 (17.1)

Physical inactivity	135,416 (44.8)	30,983 (59.8)	158, 058 (43.4)	35,651 (59.6)	179,897 (44.0)	33,423 (56.9)	146,089 (46.4)	15,561 (56.4)
Excessive alcohol consumption	100,598 (33.3)	14,134 (27.9)	116,302 (32.0)	15,127 (26.9)	26,677 (25.6)	114,573 (28.0)	90,718 (28.8)	7,733 (23.5)
Smoking	57,124 (18.9)	14,694 (18.8)	57,125 (15.7)	11,915 (15.7)	52,680 (12.9)	16,922 (13.6)	5,827 (14.2)	38,460 (12.2)
Depression	13,151 (4.4)	4,197 (6.7)	16,704 (4.6)	4,989 (6.8)	18,774 (4.6)	4,978 (4.9)	19,224 (6.1)	2,959 (8.5)
Social isolation	116.325 (38.5)	25,481 (45.7)	152,864 (42.1)	27,954 (49.1)	180,195 (44.0)	45,138 (47.1)	139,483 (44.3)	16,463 (51.6)

Abbreviations: SD: standard deviation

Table S4. Population attributable fractions for seven modifiable risk factors for dementia in the Netherlands in 2012.

	Prevalence	Communality	Relative risk[1] [95% CI]	PAF unweighted [95% CI]	PAF weighted [95% CI]
**Early-life (<45 years)**					
Low educational attainment	18·3%	34·7%	1·6 [1·3-2·0]	9·7% [4·5-15·6]	4·8% [2·4-7·1]
**Mid-life (45-65 years)**					
Obesity	15·1%	33·1%	1·3 [1·0-1·7]	4·5% [0·3-9·3]	2·2% [0·2-4·2]
Physical inactivity	40·0%	34·4%	1·3 [1·2-1·3]	9·1% [7·1-10·4]	4·4% [3·7-4·7]
Excessive alcohol consumption	36·4%	46·4%	1·2 [1·0-1·5]	7·4% [0·4-14·9]	3·6% [0·2-6·7]
Smoking	22·6%	56·3%	1·3 [1·2-1·5]	6·4% [4·0-9·2]	3·1% [2·0-4·2]
Depression	5·2%	46·8%	2·3 [1·7-3·0]	6·1% [3·5-9·3]	3·0% [1·8-4·2]
**Late-life (>65 years)**					
Social isolation	44·2%	48·4%	1·6 [1·3-1·9]	20·1% [12·4-27·3]	9·8% [6·5-12·3]
**Total PAF**					30·9% [16·8-43·4]

[1]Relative risks retrieved from the Dementia Prevention, Intervention, and Care: 2024 report of the *Lancet* Commission. [1] Abbreviations: CI: confidence interval; PAF: population attributable fraction.

Table S5. Population attributable fractions for seven modifiable risk factors for dementia in the Netherlands in 2016.

	Prevalence	Communality	Relative risk[1] [95% CI]	PAF unweighted [95% CI]	PAF weighted [95% CI]
**Early-life (<45 years)**					
Low educational attainment	12·6%	34·7%	1·6 [1·3-2·0]	6·9% [3·2-11·2]	3·4% [1·6-5·1]
**Mid-life (45-65 years)**					
Obesity	16·5%	30·9%	1·3 [1·0-1·7]	4·9% [0·3-10·1]	2·4% [0·2-4·6]
Physical inactivity	38·5%	35·6%	1·3 [1·2-1·3]	8·8% [6·8-10·0]	4·3% [3·5-4·5]
Excessive alcohol consumption	31·5%	49·1%	1·2 [1·0-1·5]	6·5% [0·3-13·1]	3·2% [0·2-6·0]
Smoking	20·3%	55·6%	1·3 [1·2-1·5]	5·7% [3·5-8·4]	2·8% [1·8-3·8]
Depression	6·4%	45·6%	2·3 [1·7-3·0]	7·4% [4·2-11·2]	3·6% [2·2-5·1]
**Late-life (>65 years)**					
Social isolation	47·5%	48·5%	1·6 [1·3-1·9]	21·3% [13·2-28·7]	10·4% [6·8-13·0]
**Total PAF**					29·9% [16·4-42·1]

[1]Relative risks retrieved from the Dementia Prevention, Intervention, and Care: 2024 report of the *Lancet* Commission. [1] Abbreviations: CI: confidence interval; PAF: population attributable fraction.

Table S6. Population attributable fractions for seven modifiable risk factors for dementia in the Netherlands in 2020.

	Prevalence	Communality	Relative risk[1] [95% CI]	PAF unweighted [95% CI]	PAF weighted [95% CI]
**Early-life (<45 years)**					
Low educational attainment	11·0%	38·4%	1·6 [1·3-2·0]	6·1% [2·8-10·0]	3·1% [1·5-4·6]
**Mid-life (45-65 years)**					
Obesity	18·4%	32·5%	1·3 [1·0-1·7]	5·4% [0·4-11·1]	2·7% [0·2-5·1]
Physical inactivity	41·0%	29·1%	1·3 [1·2-1·3]	9·3% [7·2-10·6]	4·6% [3·9-4·9]
Excessive alcohol consumption	26·4%	52·0%	1·2 [1·0-1·5]	5·5% [0·3-11·3]	2·7% [0·1-5·2]
Smoking	16·1%	54·3%	1·3 [1·2-1·5]	4·6% [2·8-6·8]	2·3% [1·5-3·1]
Depression	5·8%	47·2%	2·3 [1·7-3·0]	6·8% [3·9-10·3]	3·4% [2·1-4·8]
**Late-life (>65 years)**					
Social isolation	48·8%	46·5%	1·6 [1·3-1·9]	21·8% [13·5-29·3]	10·9% [7·3-13·6]
**Total PAF**					29·7% [16·7-41·4]

[1]Relative risks retrieved from the Dementia Prevention, Intervention, and Care: 2024 report of the *Lancet* Commission. [1] Abbreviations: CI: confidence interval; PAF: population attributable fraction.

Table S7. Population attributable fractions for seven modifiable risk factors for dementia in the Netherlands in 2022.

	Prevalence	Communality	Relative risk[1] [95% CI]	PAF unweighted [95% CI]	PAF weighted [95% CI]
**Early-life (<45 years)**					
Low educational attainment	11·5%	35·8%	1·6 [1·3-2·0]	6·4% [2·9-10·4]	3·1% [1·6-4·8]
**Mid-life (45-65 years)**					
Obesity	18·9%	32·4%	1·3 [1·0-1·7]	5·5% [0·4-11·4]	2·7% [0·2-5·2]
Physical inactivity	44·5%	31·2%	1·3 [1·2-1·3]	10·0% [7·8-11·4]	4·9% [4·2-5·2]
Excessive alcohol consumption	26·5%	53·0%	1·2 [1·0-1·5]	5·5% [0·3-11·3]	2·7% [0·1-5·1]
Smoking	15·5%	53·6%	1·3 [1·2-1·5]	4·5% [2·7-6·5]	2·2% [1·5-3·0]
Depression	8·3%	47·6%	2·3 [1·7-3·0]	9·4% [5·4-14·1]	4·6% [2·9-6·4]
**Late-life (>65 years)**					
Social isolation	49·1%	46·4%	1·6 [1·3-1·9]	21·9% [13·6-29·4]	10·8% [7·3-13·4]
**Total PAF**					31·1% [17·7-43·2]

[1]Relative risks retrieved from the Dementia Prevention, Intervention, and Care: 2024 report of the *Lancet* Commission. [1] Abbreviations: CI: confidence interval; PAF: population attributable fraction.

Table S8. Total and year-specific population attributable fractions for modifiable dementia risk factors in the Netherlands, stratified by sex.

	2012		2016		2020		2022	
	Female [95% CI]	*Male* [95% CI]	*Female* [95% CI]	*Male* [95% CI]	*Female* [95% CI]	*Male* [95% CI]	*Female* [95% CI]	*Male* [95% CI]
**Early-life (<45 years)**								
Low educational attainment	4·5% [2·2-6·7]	5·1% [2·7-7·4]	3·2% [1·5-4·9]	3·6% [1·9-5·4]	2·9% [1·4-4·4]	3·3% [1·7-4·9]	3·0% [1·5-4·6]	3·3% [1·7-4·9]
**Mid-life (45-65 years)**								
Obesity	2·3% [0·2-4·4]	2·1% [0·2-4·0]	2·5% [0·2-4·9]	2·3% [0·2-4·3]	2·9% [0·2-5·6]	2·5% [0·2-4·7]	2·9% [0·2-5·6]	2·6% [0·2-4·8]
Physical inactivity	4·4% [3·6-4·7]	4·5% [3·9-12·1]	4·2% [3·5-4·6]	4·4% [3·8-4·6]	4·6% [3·8-5·0]	4·7% [4·1-4·9]	4·9% [4·1-5·3]	5·0% [4·4-5·2]
Excessive alcohol consumption	2·6% [0·1-5·1]	4·6% [0·3-8·2]	2·2% [0·1-4·3]	4·1% [0·2-7·5]	1·8% [0·1-3·6]	3·6% [0·2-6·6]	1·8% [0·1-3·5]	3·6% [0·2-6·6]
Smoking	2·8% [1·8-3·8]	3·4% [2·4-4·5]	2·5% [1·6-3·5]	3·2% [2·2-4·2]	2·0% [1·3-2·8]	2·6% [1·8-3·5]	1·9% [1·2-3·5]	2·5% [1·8-3·4]
Depression	3·4% [2·0-4·8]	2·6% [1·6-3·6]	3·8% [2·3-5·5]	3·5% [2·2-4·8]	3·7% [2·2-5·3]	3·1% [1·9-4·3]	5·2% [3·2-7·2]	4·1% [2·6-5·6]
**Late-life (>65 years)**								
Social isolation	10·0% [2·0-12·7]	9·7% [6·7-12·1]	10·5% [6·8-13·3]	10·5% [7·3-12·9]	11·0% [7·2-13·9]	10·8% [7·5-13·3]	10·7% [7·0-13·5]	11·0% [7·7-13·4]
**Total PAF**	30·0% [16·5-42·2]	32·0% [17·7-44·7]	29·1% [16·1-41·0]	31·5% [17·8-43·7]	29·0% [16·3-40·5]	30·5% [17·4-42·2]	30·4% [17·3-42·3]	32·1% [18·5-44·0]

Abbreviations: CI: confidence interval; PAF: population attributable fraction.

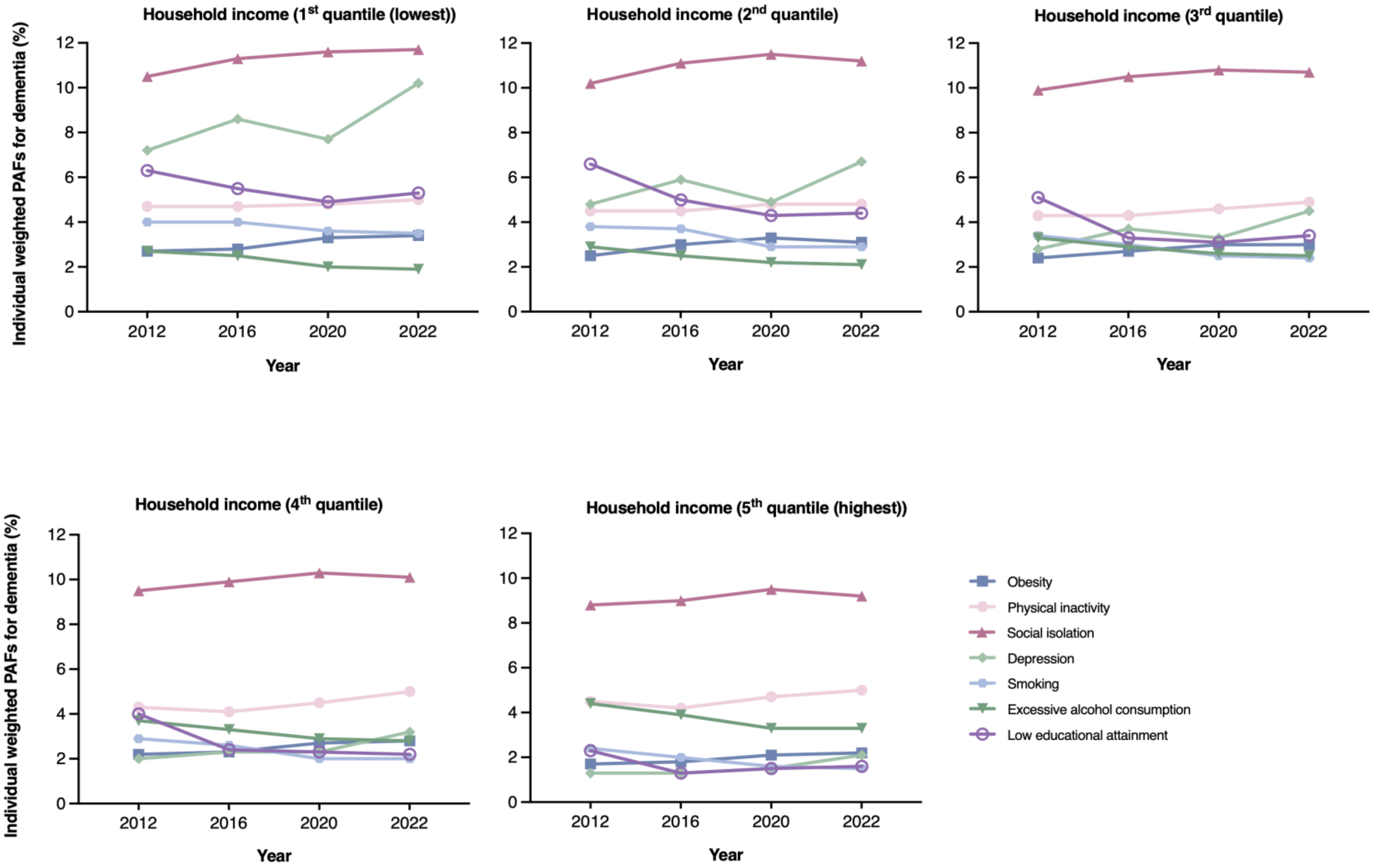

Figure S3: Weighted population attributable fractions for individual modifiable dementia risk factors, stratified by household income. ***Abbreviation: PAF: population attributable fraction.***

Table S9. Total population attributable fractions for modifiable dementia risk factors in the Netherlands in 2012, stratified by household income.

	2012				
	1st household income quantile [95% CI]	*2nd household income quantile* [95% CI]	*3rd household income quantile* [95% CI]	*4th household income quantile* [95% CI]	*5th household income quantile* [95% CI]
**Early-life (<45 years)**					
Low educational attainment	6·3% [3·2-9·0]	6·6% [3·4-9·5]	5·1% [2·6-7·5]	4·0% [2·0-5·9]	2·3% [1·1-3·5]
**Mid-life (45-65 years)**					
Obesity	2·7% [0·2-5·0]	2·5% [0·2-4·8]	2·4% [0·2-4·6]	2·2% [0·2-4·2]	1·7% [0·1-3·4]
Physical inactivity	4·7% [3·9-4·9]	4·5% [3·7-4·7]	4·3% [3·7-4·6]	4·3% [3·6-4·5]	4·5% [3·8-4·8]
Excessive alcohol consumption	2·7% [0·1-5·0]	2·9% [0·1-5·3]	3·3% [0·2-6·2]	3·7% [0·2-6·9]	4·4% [0·2-8·0]
Smoking	4·0% [2·7-5·3]	3·8% [2·5-5·0]	3·4% [2·3-4·5]	2·9% [2·0-4·0]	2·4% [1·6-3·3]
Depression	7·2% [4·6-9·6]	4·8% [3·0-6·5]	2·8% [1·7-4·0]	2·0% [1·2-2·8]	1·3% [0·8-1·9]
**Late-life (>65 years)**					
Social isolation	10·5% [7·0-12·9]	10·2% [6·8-12·6]	9·9% [6·6-12·4]	9·5% [6·3-12·0]	8·8% [5·8-11·2]
**Total PAF**	38·0% [21·7-51·8]	35·2% [19·7-48·5]	31·3% [17·2-43·8]	28·5% [15·4-40·3]	25·4% [13·5-36·2]

Abbreviations: CI: confidence interval; PAF: population attributable fraction.

Table S10. Total population attributable fractions for modifiable dementia risk factors in the Netherlands in 2016, stratified by household income.

	2016				
	1st household income quantile [95% CI]	*2nd household income quantile* [95% CI]	*3rd household income quantile* [95% CI]	*4th household income quantile* [95% CI]	*5th household income quantile* [95% CI]
**Early-life (<45 years)**					
Low educational attainment	5·5% [2·8-7·9]	5·0% [2·5-7·3]	3·3% [1·6-5·1]	2·4% [1·2-3·7]	1·3% [0·6-2·1]
**Mid-life (45-65 years)**					
Obesity	2·8% [0·2-5·2]	3·0% [0·2-5·7]	2·7% [0·2-5·2]	2·3% [0·2-4·5]	1·8% [0·1-3·6]
Physical inactivity	4·7% [3·9-4·9]	4·5% [3·8-4·7]	4·3% [3·6-4·5]	4·1% [3·5-4·4]	4·2% [3·5-4·5]
Excessive alcohol consumption	2·2% [0·1-4·2]	2·5% [0·1-4·8]	2·9% [0·2-5·5]	3·3% [0·2-6·2]	3·9% [0·2-7·3]
Smoking	4·0% [2·7-5·3]	3·7% [2·5-4·8]	3·0% [2·0-4·1]	2·6% [1·7-3·5]	2·0% [1·3-2·8]
Depression	8·6% [5·5-11·3]	5·9% [3·7-8·0]	3·7% [2·3-5·2]	2·3% [1·4-3·3]	1·3% [0·8-1·9]
**Late-life (>65 years)**					
Social isolation	11·3% [7·6-13·8]	11·1% [7·4-13·7]	10·5% [7·0-13·2]	9·9% [6·6-12·5]	9·0% [5·9-11·6]
**Total PAF**	39·0% [22·7-52·7]	35·7% [20·2-49·1]	30·5% [16·8-42·8]	26·9% [14·6-38·1]	23·5% [12·4-33·8]

Abbreviations: CI: confidence interval; PAF: population attributable fraction.

Table S11. Total population attributable fractions for modifiable dementia risk factors in the Netherlands in 2020, stratified by household income.

	2020				
	1st household income quantile [95% CI]	*2nd household income quantile* [95% CI]	*3rd household income quantile* [95% CI]	*4th household income quantile* [95% CI]	*5th household income quantile* [95% CI]
**Early-life (<45 years)**					
Low educational attainment	4·9% [2·5-7·2]	4·3% [2·2-6·4]	3·1% [1·5-4·8]	2·3% [1·1-3·6]	1·5% [0·7-2·3]
**Mid-life (45-65 years)**					
Obesity	3·3% [0·2-6·1]	3·3% [0·2-6·2]	3·0% [0·2-5·6]	2·7% [0·2-5·1]	2·1% [0·2-4·1]
Physical inactivity	4·8% [4·0-5·1]	4·8% [4·0-5·0]	4·6% [3·9-4·9]	4·5% [3·8-4·8]	4·7% [4·0-5·0]
Excessive alcohol consumption	2·0% [0·1-3·7]	2·2% [0·1-4·2]	2·6% [0·1-5·0]	2·9% [0·2-5·5]	3·3% [0·2-6·3]
Smoking	3·6% [2·4-4·8]	2·9% [2·0-3·9]	2·5% [1·6-3·4]	2·0% [1·4-2·8]	1·6% [1·1-2·2]
Depression	7·7% [4·9-10·2]	4·9% [3·1-6·8]	3·3% [1·6-4·7]	2·3% [1·4-3·2]	1·5% [0·9-2·2]
**Late-life (>65 years)**					
Social isolation	11·6% [7·8-14·1]	11·5% [7·7-14·1]	10·8% [7·2-13·5]	10·3% [6·9-13·0]	9·5% [6·4-12·1]
**Total PAF**	37·8% [22·0-51·3]	34·0% [19·3-46·8]	30·0% [16·7-41·9]	27·0% [15·0-38·0]	24·2% [13·5-34·1]

Abbreviations: CI: confidence interval; PAF: population attributable fraction.

Table S12. Total population attributable fractions for modifiable dementia risk factors in the Netherlands in 2022, stratified by household income.

	2022				
	1st household income quantile [95% CI]	*2nd household income quantile* [95% CI]	*3rd household income quantile* [95% CI]	*4th household income quantile* [95% CI]	*5th household income quantile* [95% CI]
**Early-life (<45 years)**					
Low educational attainment	5·3% [2·7-7·7]	4·4% [2·2-6·6]	3·4% [1·7-5·1]	2·2% [1·1-3·3]	1·6% [0·8-2·5]
**Mid-life (45-65 years)**					
Obesity	3·4% [0·3-6·3]	3·1% [0·2-5·9]	3·0% [0·2-5·6]	2·8% [0·2-5·3]	2·2% [0·2-4·2]
Physical inactivity	5·0% [4·2-5·2]	4·8% [3·9-5·0]	4·9% [4·2-5·2]	5·0% [4·3-5·3]	5·0% [4·3-5·3]
Excessive alcohol consumption	1·9% [0·1-3·6]	2·1% [0·1-4·0]	2·5% [0·1-4·8]	2·8% [0·1-5·3]	3·3% [0·2-6·3]
Smoking	3·5% [2·3-4·6]	2·9% [1·9-3·8]	2·4% [1·6-3·2]	2·0% [1·3-2·7]	1·5% [1·0-2·0]
Depression	10·2% [6·7-13·1]	6·7% [1·9-9·0]	4·5% [2·8-6·2]	3·2% [2·0-4·6]	2·1% [1·3-3·0]
**Late-life (>65 years)**					
Social isolation	11·7% [7·9-14·2]	11·2% [7·5-13·9]	10·7% [7·3-13·3]	10·1% [6·9-12·7]	9·2% [6·2-11·7]
**Total PAF**	40·9% [24·2-54·6]	35·2% [20·0-48·3]	31·4% [17·9-43·4]	28·1% [15·9-39·1]	25·0% [13·9-35·1]

Abbreviations: CI: confidence interval; PAF: population attributable fraction.

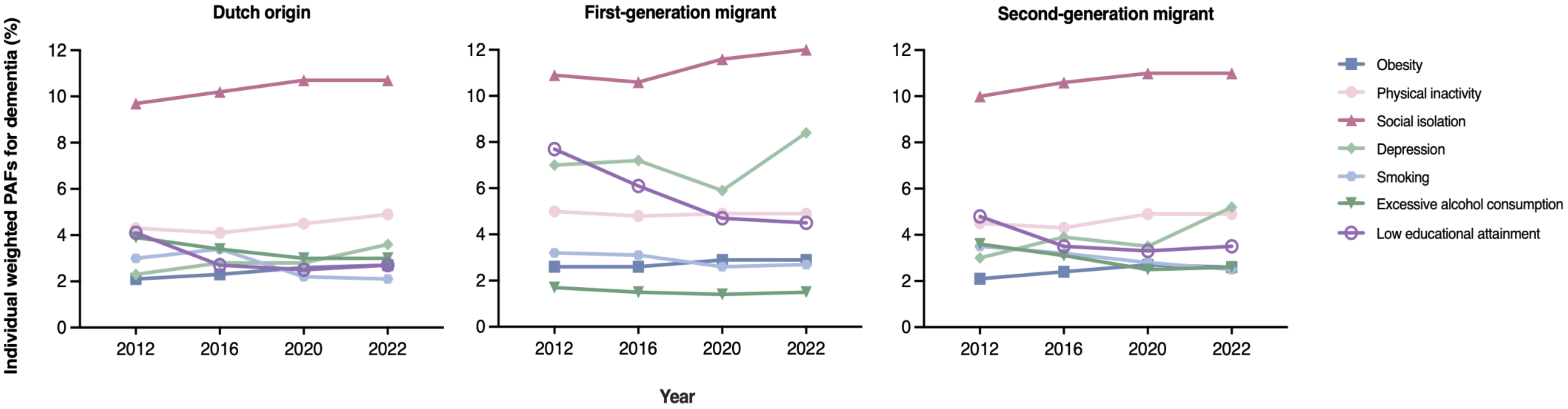

Figure S4: Weighted population attributable fractions for individual modifiable dementia risk factors, stratified by migration background. ***Abbreviation: PAF: population attributable fraction.***

Table S13. Total and population attributable fractions for modifiable dementia risk factors in the Netherlands in 2012, stratified by migration background.

	2012		
	Dutch origin [95% CI]	*1st generation migrant* [95% CI]	*2nd generation migrant* [95% CI]
**Early-life (<45 years)**			
Low educational attainment	4·1% [2·0- 6·2]	7·7% [4·0- 10·9]	4·8% [2·4-7·1]
**Mid-life (45-65 years)**			
Obesity	2·1% [0·2-4·1]	2·6% [0·2-5·0]	2·1% [0·2-4·1]
Physical inactivity	4·3% [3·6- 4·6]	5·0% [4·2-5·3]	4·5% [3·8-4·7]
Excessive alcohol consumption	3·9% [0·2-7·2]	1·7% [0·1-3·4]	3·6% [0·2-6·6]
Smoking	3·0% [2·0-4·1]	3·2% [2·1-4·3]	3·5% [2·3-4·6]
Depression	2·3% [1·4-3·2]	7·0% [4·4-9·4]	3·0% [1·8-4·2]
**Late-life (>65 years)**			
Social isolation	9·7% [6·4-12·3]	10·9% [7·3-13·5]	10·0% [6·7-12·5]
**Total PAF**	29·4% [15·7-41·7]	38·2% [22·3-51·8]	31·4% [17·4-43·9]

Abbreviations: CI: confidence interval; PAF: population attributable fraction.

Table S14. Total and population attributable fractions for modifiable dementia risk factors in the Netherlands in 2016, stratified by migration background.

	2016		
	Dutch origin [95% CI]	*1st generation migrant* [95% CI]	*2nd generation migrant* [95% CI]
**Early-life (<45 years)**			
Low educational attainment	2·7% [1·3-4·2]	6·1% [3·1-8·9]	3·5% [1·7-5·3]
**Mid-life (45-65 years)**			
Obesity	2·3% [0·2-4·5]	2·6% [0·2-5·0]	2·4% [0·2-4·6]
Physical inactivity	4·1% [3·4-4·5]	4·8% [4·0-5·1]	4·3% [3·6-4·6]
Excessive alcohol consumption	3·4% [0·2-6·4]	1·5% [0·1-3·0]	3·1% [0·2-5·8]
Smoking	2·7% [1·8-3·7]	3·1% [2·1-4·2]	3·2% [2·1-4·3]
Depression	2·8% [1·7-4·1]	7·2% [4·5-9·7]	3·9% [2·4-5·5]
**Late-life (>65 years)**			
Social isolation	10·2% [6·7-12·9]	11·3% [7·5-14·0]	10·6% [7·0-13·3]
**Total PAF**	28·3% [15·3-40·2]	36·7% [21·5-49·9]	30·9% [17·0-43·4]

Abbreviations: CI: confidence interval; PAF: population attributable fraction.

Table S15. Total and population attributable fractions for modifiable dementia risk factors in the Netherlands in 2020, stratified by migration background.

	2020		
	Dutch origin [95% CI]	*1st generation migrant* [95% CI]	*2nd generation migrant* [95% CI]
**Early-life (<45 years)**			
Low educational attainment	2·5% [1·2-3·9]	4·7% [2·3-6·9]	3·3% [1·6-4·9]
**Mid-life (45-65 years)**			
Obesity	2·6% [0·2-5·0]	2·9% [0·2-5·6]	2·7% [0·2-5·1]
Physical inactivity	4·5% [3·8-4·8]	4·9% [4·1-5·1]	4·9% [4·1-5·1]
Excessive alcohol consumption	3·0% [0·2-5·7]	1·4% [0·1-2·7]	2·5% [0·1-4·7]
Smoking	2·2% [1·4-3·0]	2·6% [1·7-3·5]	2·8% [1·9-3·8]
Depression	2·8% [1·7-4·0]	5·9% [3·7-8·0]	3·5% [2·2-5·0]
**Late-life (>65 years)**			
Social isolation	10·7% [7·2-13·5]	11·6% [7·9-14·3]	11·0% [7·5-13·7]
**Total PAF**	28·4% [15·7-39·9]	34·0% [20·0-46·2]	30·6% [17·6-42·3]

Abbreviations: CI: confidence interval; PAF: population attributable fraction.

Table S16. Total and population attributable fractions for modifiable dementia risk factors in the Netherlands in 2022, stratified by migration background.

	2022		
	Dutch origin [95% CI]	*1st generation migrant* [95% CI]	*2nd generation migrant* [95% CI]
**Early-life (<45 years)**			
Low educational attainment	2·7 [1·3-4·1]	4·5 [2·2-6·6]	3·5 [1·8-5·3]
**Mid-life (45-65 years)**			
Obesity	2·7 [0·2-5·2]	2·9 [0·2-5·6]	2·6 [0·2-5·0]
Physical inactivity	4·9 [4·2-5·2]	4·9 [4·1-5·2]	4·9 [4·1-5·1]
Excessive alcohol consumption	3·0 [0·2-5·6]	1·5 [0·1-2·9]	2·6 [0·1-4·9]
Smoking	2·1 [1·4-5·6]	2·7 [1·8-3·6]	2·5 [1·6-3·3]
Depression	3·6 [2·3-5·1]	8·4 [5·4-11·2]	5·2 [3·3-7·1]
**Late-life (>65 years)**			
Social isolation	10·7 [7·1-13·3]	12·0 [8·1-14·7]	11·0 [7·5-13·6]
**Total PAF**	29·6 [16·6-41·4]	36·9 [22·0-49·8]	32·3 [18·6-44·4]

Abbreviations: CI: confidence interval; PAF: population attributable fraction.

Table S17. Total and year-specific population attributable fractions for modifiable dementia risk factors in the Netherlands, stratified by region.

	2012 [95% CI]	2016 [95% CI]	2020 [95% CI]	2022 [95% CI])
GGD Groningen	29·6% [16·0-41·8]	29·2% [15·6-41·5]	29·0% [16·1-40·7]	30·6% [16·7-43·0]
GGD Drenthe	36·4% [20·4-50·2]	29·8% [15·9-42·3]	28·9% [15·6-40·8]	31·0% [17·0-43·5]
GGD IJsselland	29·5% [16·1-41·4]	28·0% [14·9-40·0]	29·2% [16·2-40·9]	30·4% [17·3-42·1]
GGD Twente	31·6% [17·6-43·9]	29·3% [15·8-41·5]	30·1% [16·8-42·1]	30·6% [16·7-43·0]
GGD Noord- en Oost-Gelderland	30·0% [16·6-42·0]	28·3% [15·2-40·2]	29·7% [16·1-41·8]	30·3% [17·0-42·2]
Veiligsheids- en Gezondheidsregio Gelderland-Midden	30·9% [17·3-42·9]	29·7% [16·5-41·8]	29·5% [16·4-41·3]	30·2% [17·4-41·6]
GGD Gelderland-Zuid	30·3% [16·5-42·8]	29·4% [15·9-41·6]	29·6% [16·4-41·4]	29·0% [16·0-40·8]
GGD Flevoland	33·0% [18·7-45·7]	38·7% [22·9-52·1]	31·3% [17·8-43·3]	32·2% [18·3-44·7]
GGD Regio Utrecht	29·8% [16·4-41·9]	29·1% [16·2-40·8]	27·7% [15·7-38·5]	28·9% [16·5-40·1]
GGD Hollands-Noorden	30·5% [16·2-43·2]	29·4% [16·1-41·4]	28·5% [15·7-40·1]	30·5% [17·0-42·7]
GGD Kennermerland	30·3% [16·1-42·9]	29·3% [15·8-41·5]	28·2% [15·6-39·7]	30·0% [16·8-42·0]
GGD Amsterdam	37·7% [20·9-51·9]	31·0% [17·4-43·3]	29·3% [16·8-40·6]	33·8% [19·6-46·2]
GGD Gooi en Vechtstreek	30·9% [16·7-43·4]	28·9% [15·7-40·8]	27·8% [15·6-38·6]	30·1% [17·4-41·4]
GGD Hollands-Midden	29·7% [15·7-42·3]	29·2% [16·2-41·1]	28·7% [16·6-39·7]	29·2% [16·3-40·8]
GGD Rotterdam-Rijnmond	34·1% [19·5-46·8]	33·1% [18·6-45·9]	33·1% [19·1-45·4]	33·8% [19·6-46·3]
Dienst Gezondheid & Jeugd Zuid-Holland Zuid	31·5% [17·8-43·7]	30·5% [16·9-42·7]	30·3% [17·0-42·2]	32·4% [18·6-44·5]
GGD Zeeland	30·8% [16·5-43·5]	29·8% [16·2-42·0]	30·1% [17·3-41·6]	31·8% [18·1-43·9]
GGD West-Brabant	39·0% [21·9-53·2]	30·3% [16·0-43·1]	29·4% [16·1-41·3]	32·2% [18·0-44·7]
GGD Hart voor Brabant	30·8% [16·2-43·8]	29·2% [15·5-41·6]	29·3% [16·2-41·0]	30·8% [17·3-42·9]
GGD Brabant-Zuidoost	29·7% [15·8-42·1]	27·9% [15·0-39·7]	28·8% [16·1-40·3]	31·1% [17·9-42·8]
GGD Limburg-Noord	37·2% [20·8-51·1]	37·6% [21·6-51·1]	29·4% [16·4-41·1]	37·7% [21·9-51·2]
GGD Zuid-Limburg	32·5% [17·9-45·4]	40·9% [24·0-54·9]	32·0% [17·7-44·6]	32·4% [18·0-45·2]
GGD Haaglanden	31·9% [17·5-44·7]	31·7% [17·5-44·3]	30·2% [17·3-41·7]	32·5% [18·8-44·7]
GGD Fryslân	30·6% [16·6-43·1]	28·4% [15·3-40·3]	27·3% [14·4-39·1]	30·5% [17·0-42·6]
GGD Zaanstreek/Waterland	33·4% [18·8-46·2]	30·5% [16·9-42·7]	30·4% [16·7-42·3]	32·9% [19·1-45·1]

Abbreviations: CI: confidence interval; GGD: municipal public health service

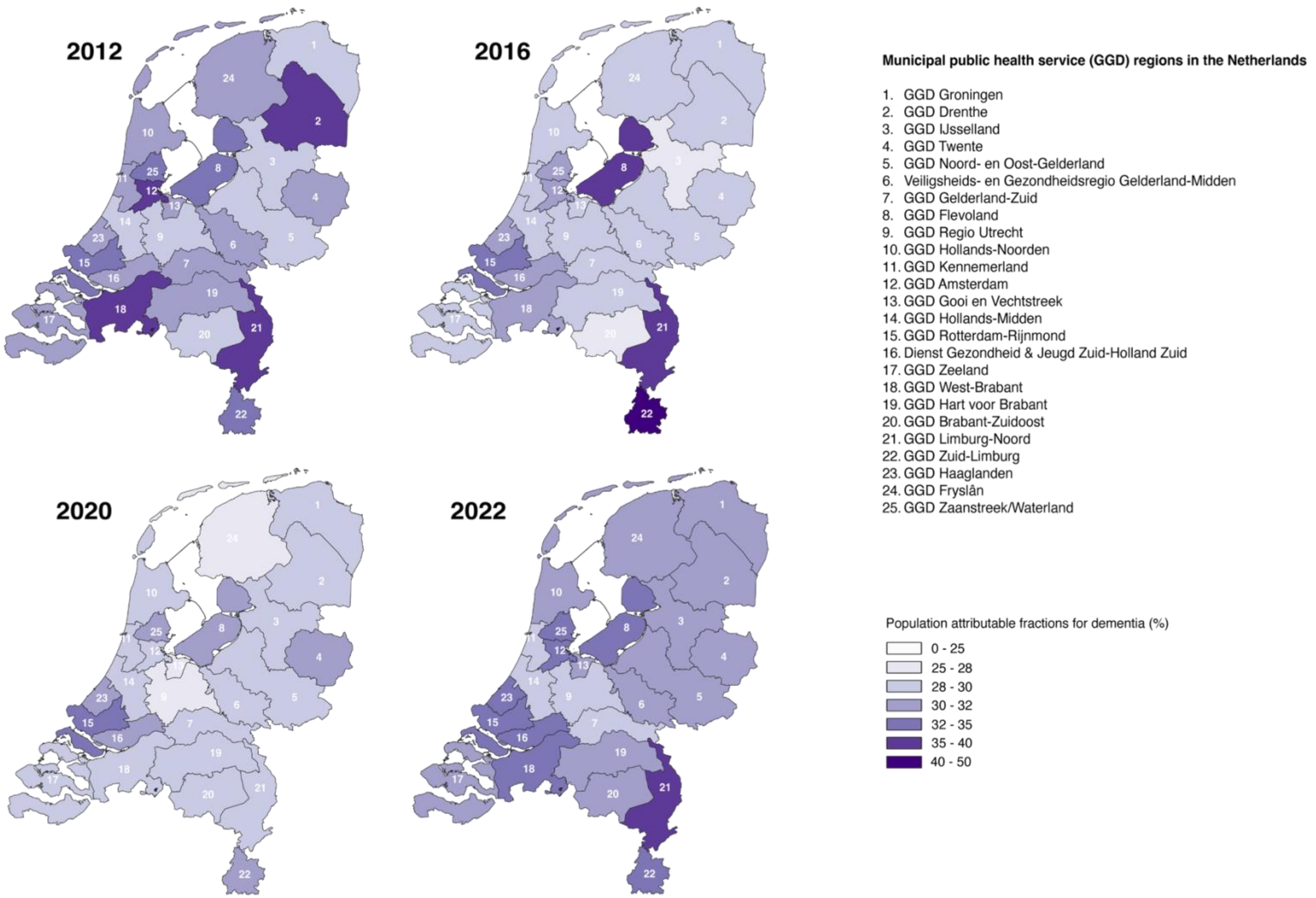

Figure S5: Choropleth maps of population attributable fractions for seven modifiable dementia risk factors across 25 municipal public health service (GGD) regions of the Netherlands, 2012-2022.

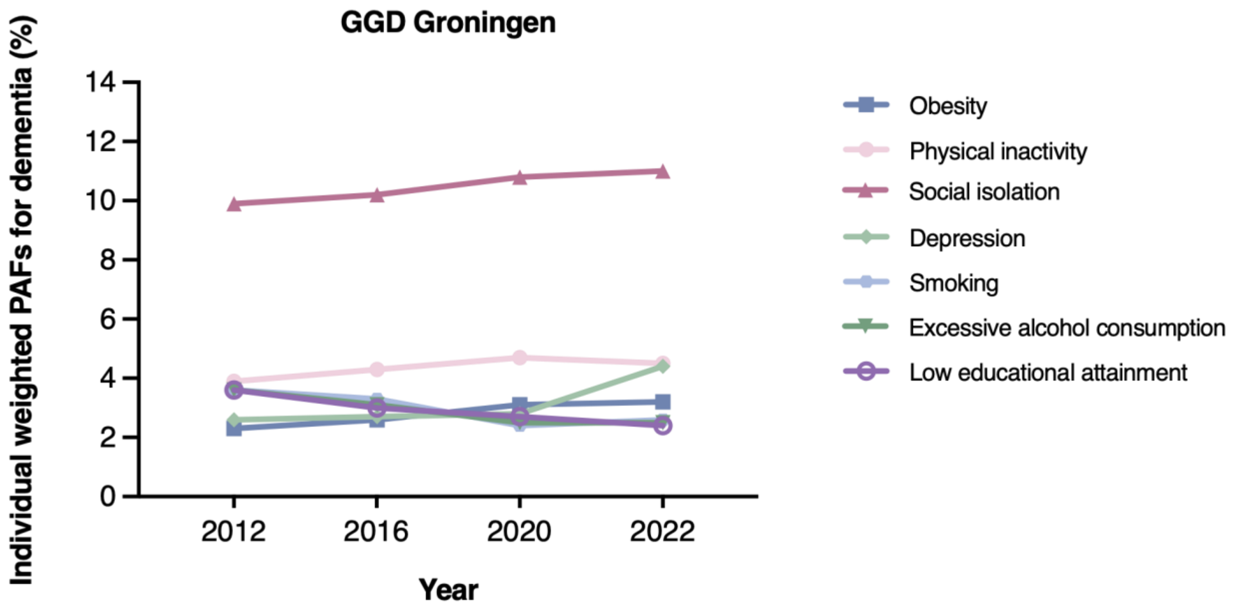

Figure S6. Individual weighted population attributable fractions of seven modifiable risk factors for dementia in municipal public health service (GGD) Groningen in 2012, 2016, 2020, and 2022. ***Abbreviation: PAF: population attributable fraction.***

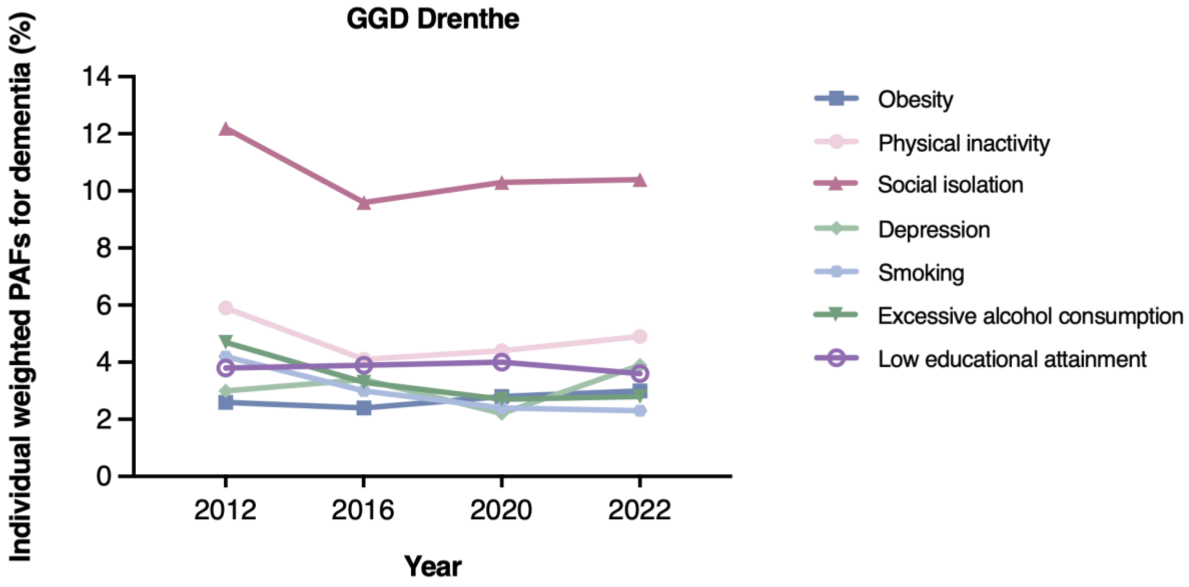

Figure S7. Individual weighted population attributable fractions of seven modifiable risk factors for dementia in municipal public health service (GGD) Drenthe in 2012, 2016, 2020, and 2022. ***Abbreviation: PAF: population attributable fraction.***

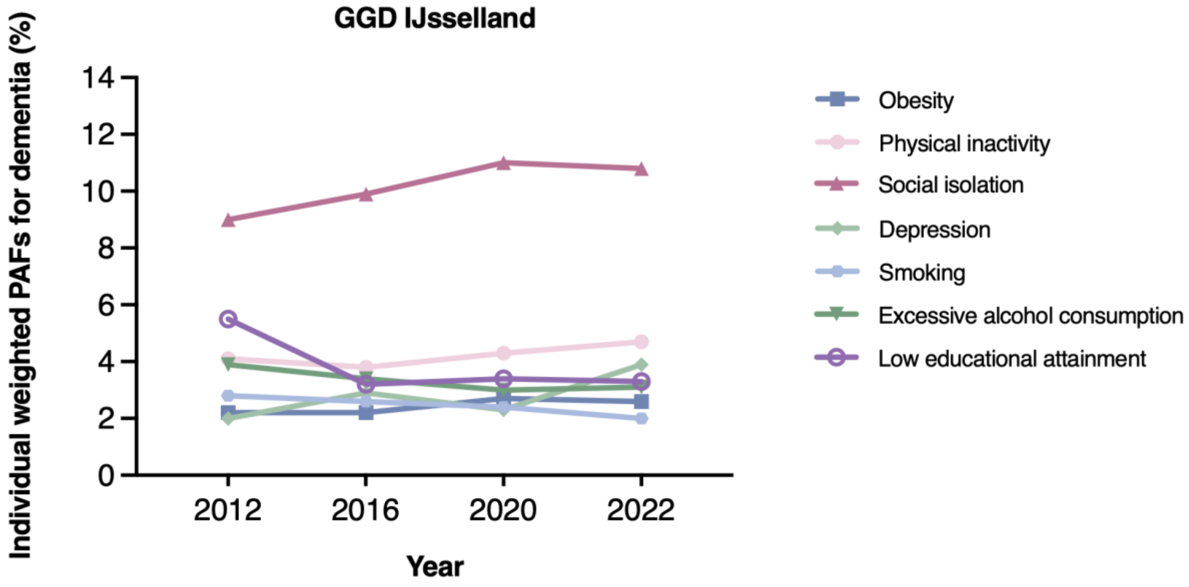

Figure S8. Individual weighted population attributable fractions of seven modifiable risk factors for dementia in municipal public health service (GGD) IJsselland in 2012, 2016, 2020, and 2022. ***Abbreviation: PAF: population attributable fraction.***

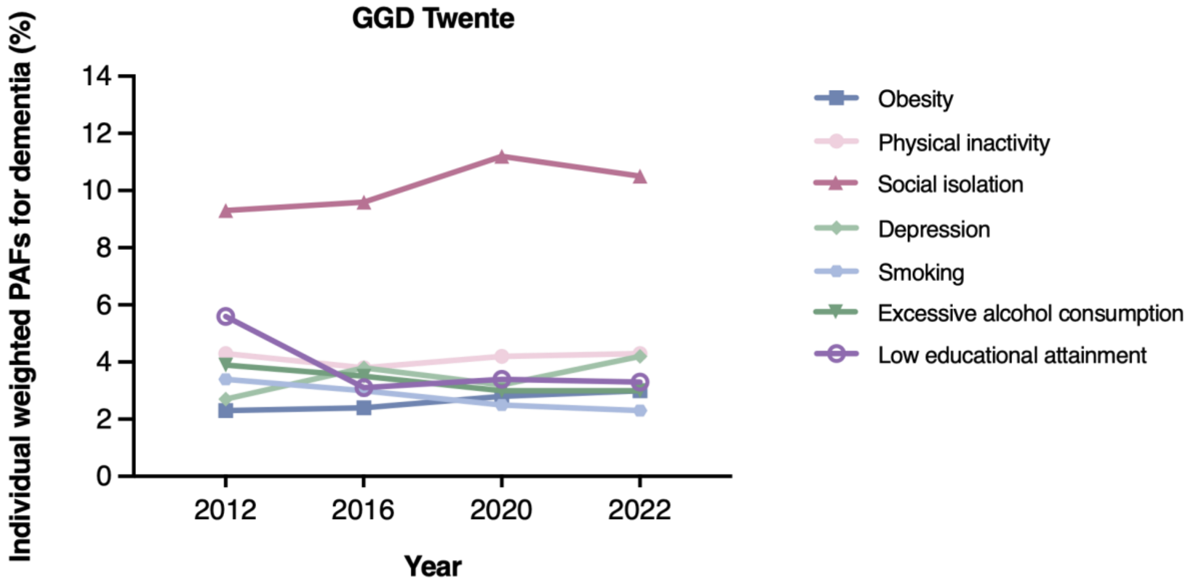

Figure S9. Individual weighted population attributable fractions of seven modifiable risk factors for dementia in municipal public health service (GGD) Twente in 2012, 2016, 2020, and 2022. ***Abbreviation: PAF: population attributable fraction.***

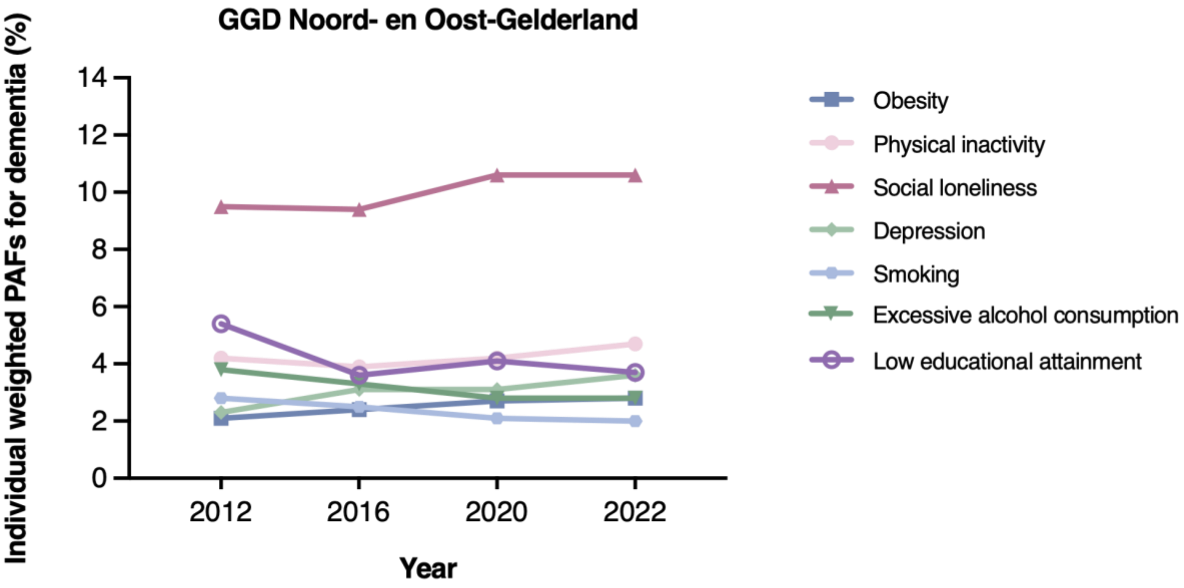

Figure S10. Individual weighted population attributable fractions of seven modifiable risk factors for dementia in municipal public health service (GGD) Noord- en Oost-Gelderland in 2012, 2016, 2020, and 2022. ***Abbreviation: PAF: population attributable fraction.***

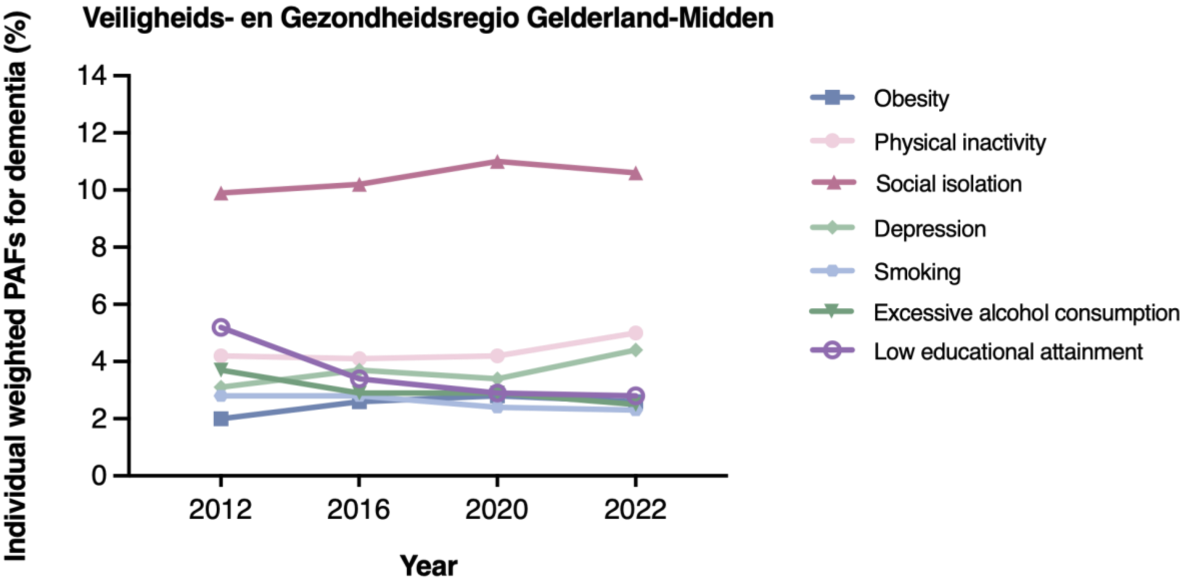

Figure S11. Individual weighted population attributable fractions of seven modifiable risk factors for dementia in Veiligheids- en Gezondheidsregio Gelderland-Midden in 2012, 2016, 2020, and 2022. ***Abbreviation: PAF: population attributable fraction.***

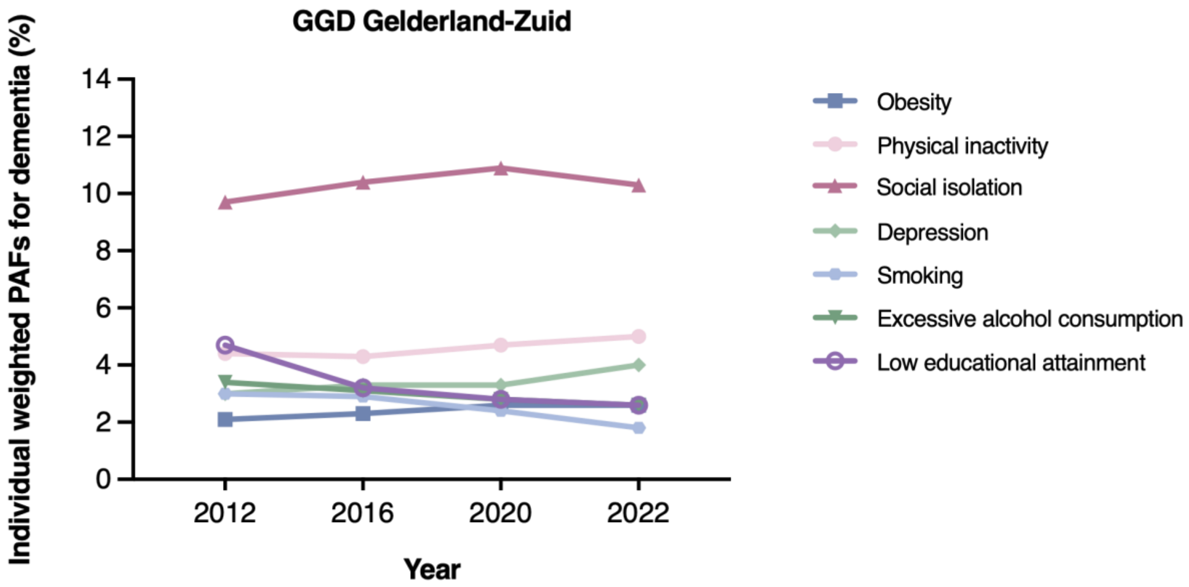

Figure S12. Individual weighted population attributable fractions of seven modifiable risk factors for dementia in municipal public health service (GGD) Gelderland-Zuid in 2012, 2016, 2020, and 2022. ***Abbreviation: PAF: population attributable fraction.***

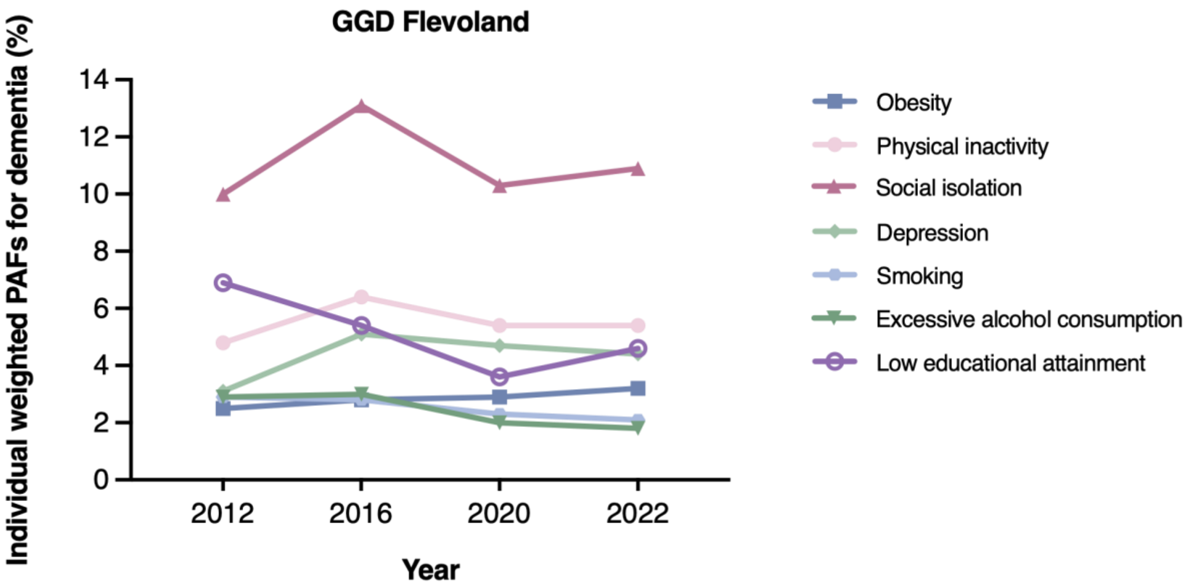

Figure S13. Individual weighted population attributable fractions of seven modifiable risk factors for dementia in municipal public health service (GGD) Flevoland in 2012, 2016, 2020, and 2022. ***Abbreviation: PAF: population attributable fraction.***

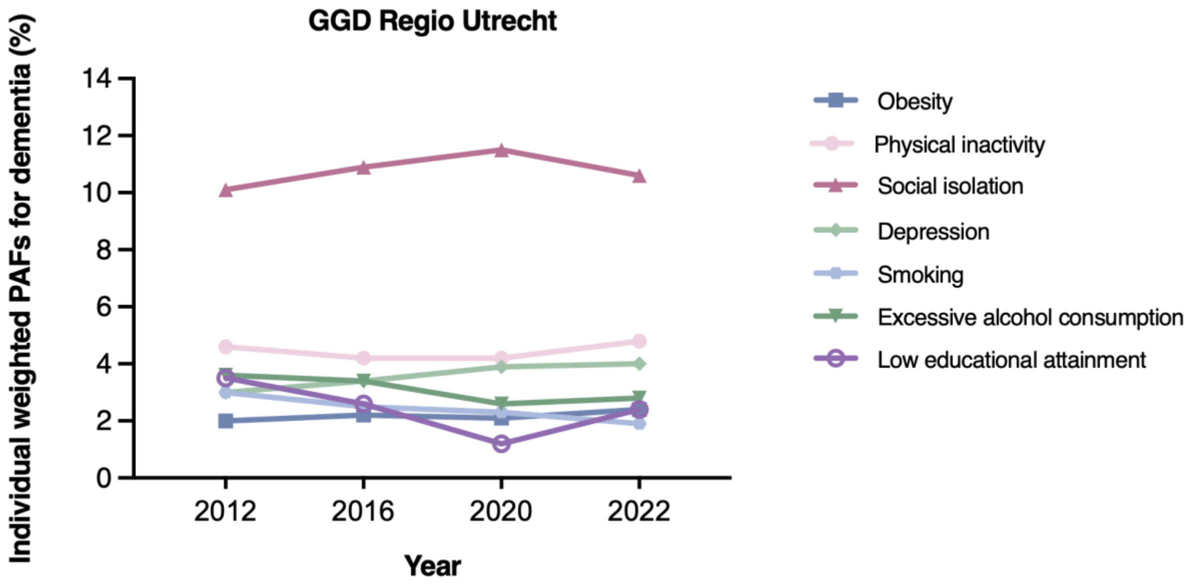

Figure S14. Individual weighted population attributable fractions of seven modifiable risk factors for dementia in municipal public health service (GGD) Regio Utrecht in 2012, 2016, 2020, and 2022.
Abbreviation: PAF: population attributable fraction.

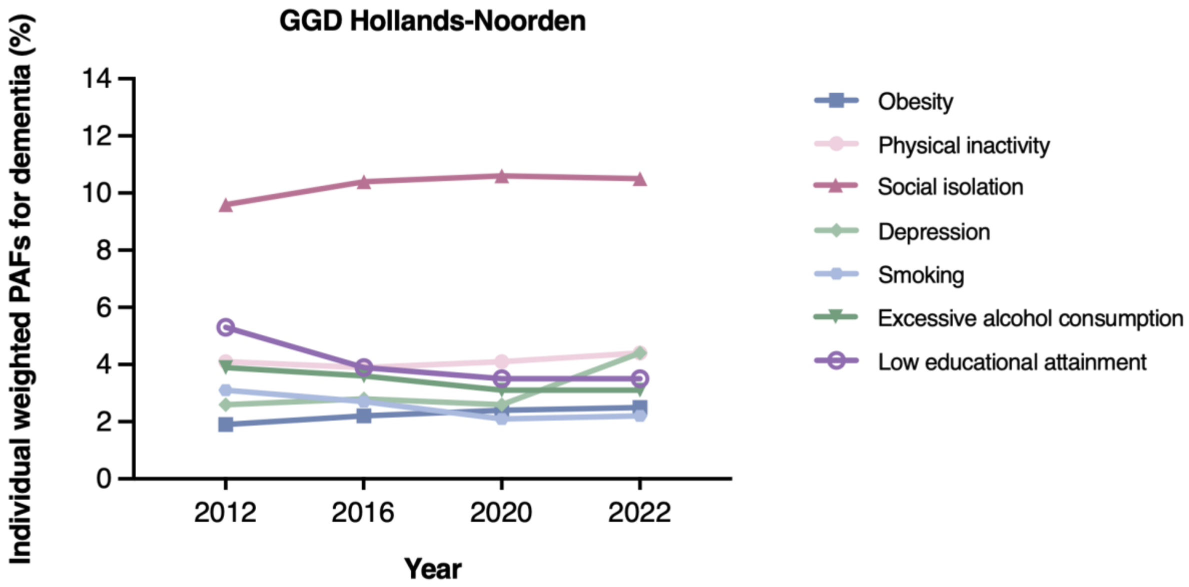

Figure S15. Individual weighted population attributable fractions of seven modifiable risk factors for dementia in municipal public health service (GGD) Hollands-Noorden in 2012, 2016, 2020, and 2022.
Abbreviation: PAF: population attributable fraction.

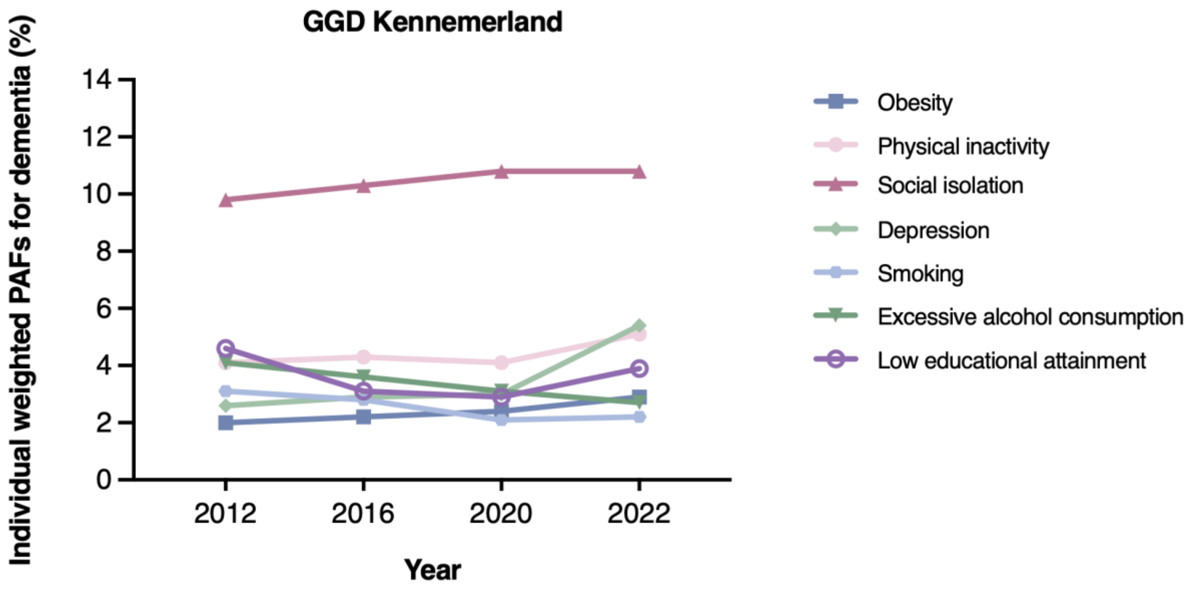

Figure S16. Individual weighted population attributable fractions of seven modifiable risk factors for dementia in municipal public health service (GGD) Kennemerland in 2012, 2016, 2020, and 2022.
Abbreviation: PAF: population attributable fraction.

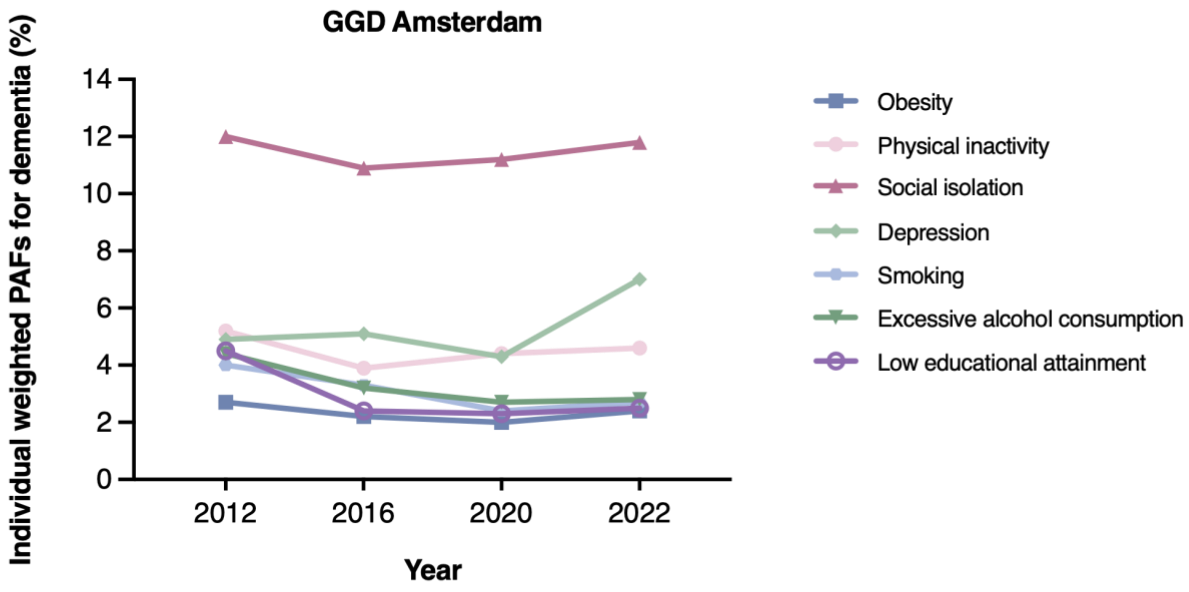

Figure S17. Individual weighted population attributable fractions of seven modifiable risk factors for dementia in municipal public health service (GGD) Amsterdam in 2012, 2016, 2020, and 2022.
Abbreviation: PAF: population attributable fraction.

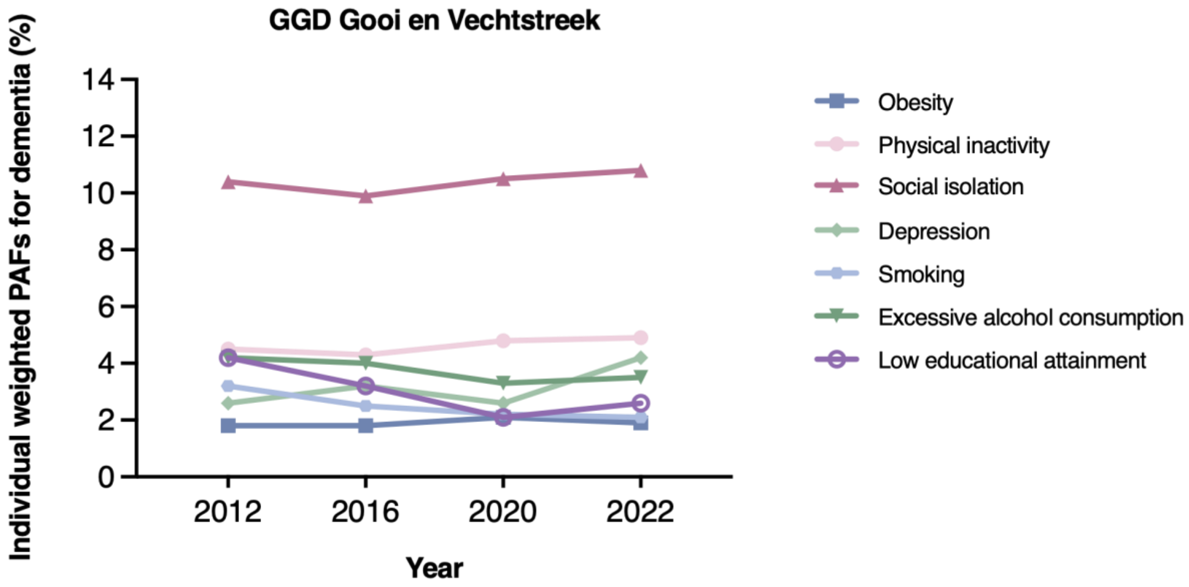

Figure S18. Individual weighted population attributable fractions of seven modifiable risk factors for dementia in municipal public health service (GGD) Gooi en Vechtstreek in 2012, 2016, 2020, and 2022.
Abbreviation: PAF: population attributable fraction.

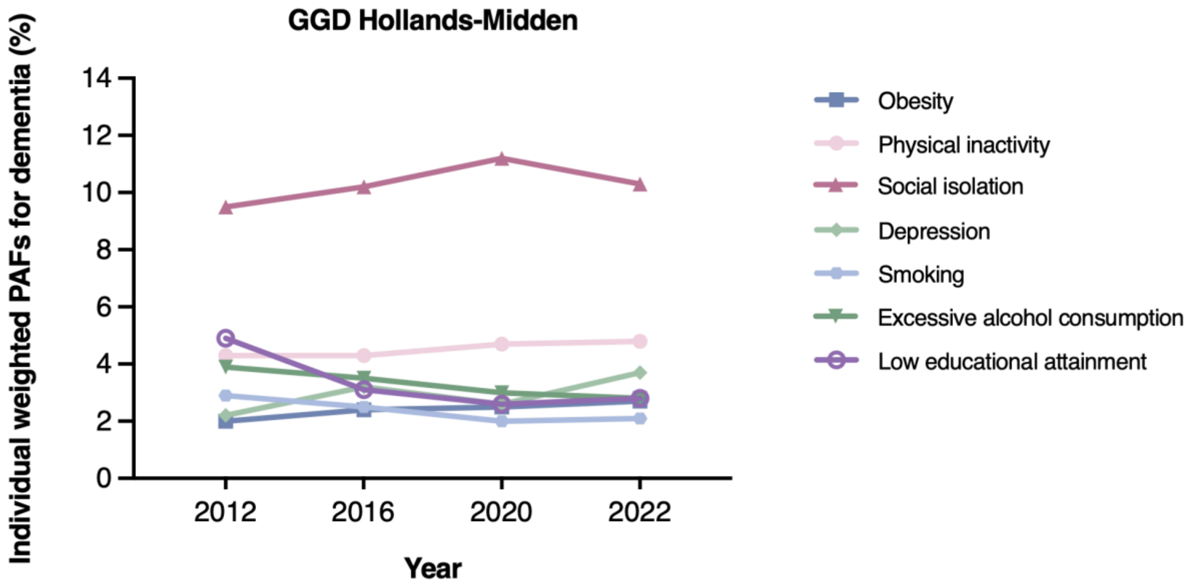

Figure S19. Individual weighted population attributable fractions of seven modifiable risk factors for dementia in municipal public health service (GGD) Hollands-Midden in 2012, 2016, 2020, and 2022.
Abbreviation: PAF: population attributable fraction.

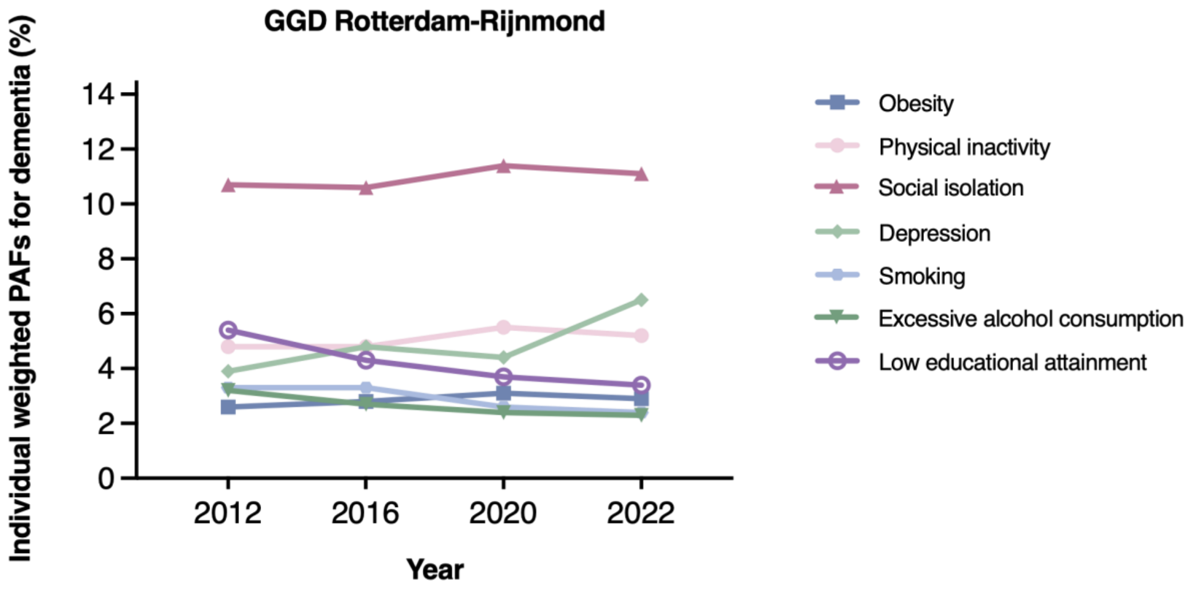

Figure S20. Individual weighted population attributable fractions of seven modifiable risk factors for dementia in municipal public health service (GGD) Rotterdam-Rijnmond in 2012, 2016, 2020, and 2022. ***Abbreviation: PAF: population attributable fraction.***

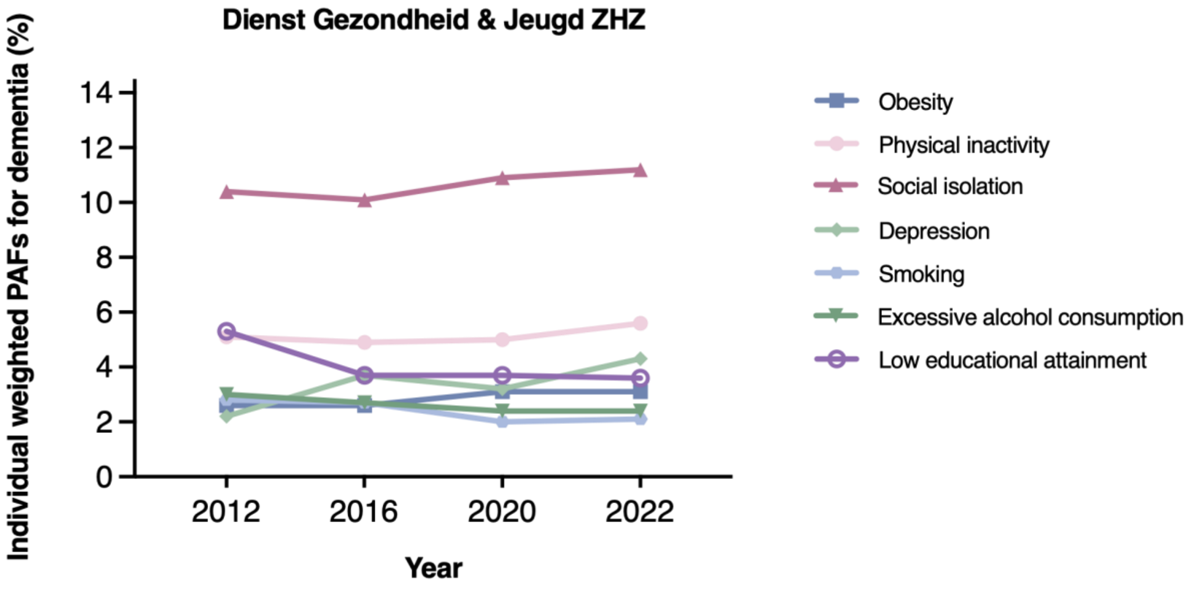

Figure S21. Individual weighted population attributable fractions of seven modifiable risk factors for dementia in Dienst Gezondheid & Jeugd Zuid-Holland Zuid in 2012, 2016, 2020, and 2022. ***Abbreviation: PAF: population attributable fraction.***

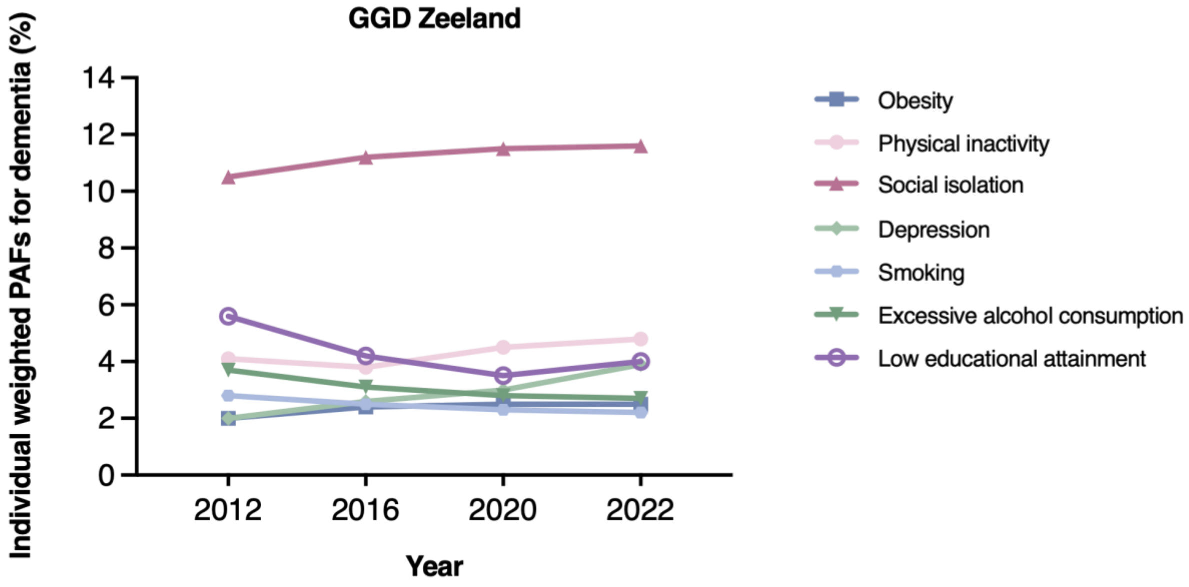

Figure S22. Individual weighted population attributable fractions of seven modifiable risk factors for dementia in municipal public health service (GGD) Zeeland in 2012, 2016, 2020, and 2022. ***Abbreviation: PAF: population attributable fraction.***

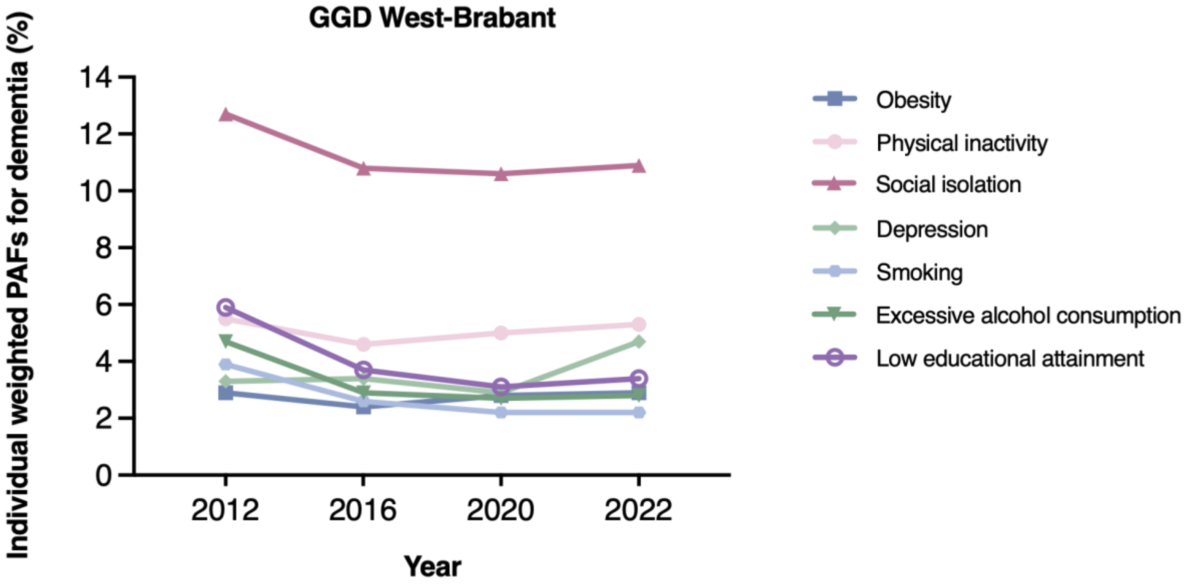

Figure S23. Individual weighted population attributable fractions of seven modifiable risk factors for dementia in municipal public health service (GGD) West-Brabant in 2012, 2016, 2020, and 2022. ***Abbreviation: PAF: population attributable fraction.***

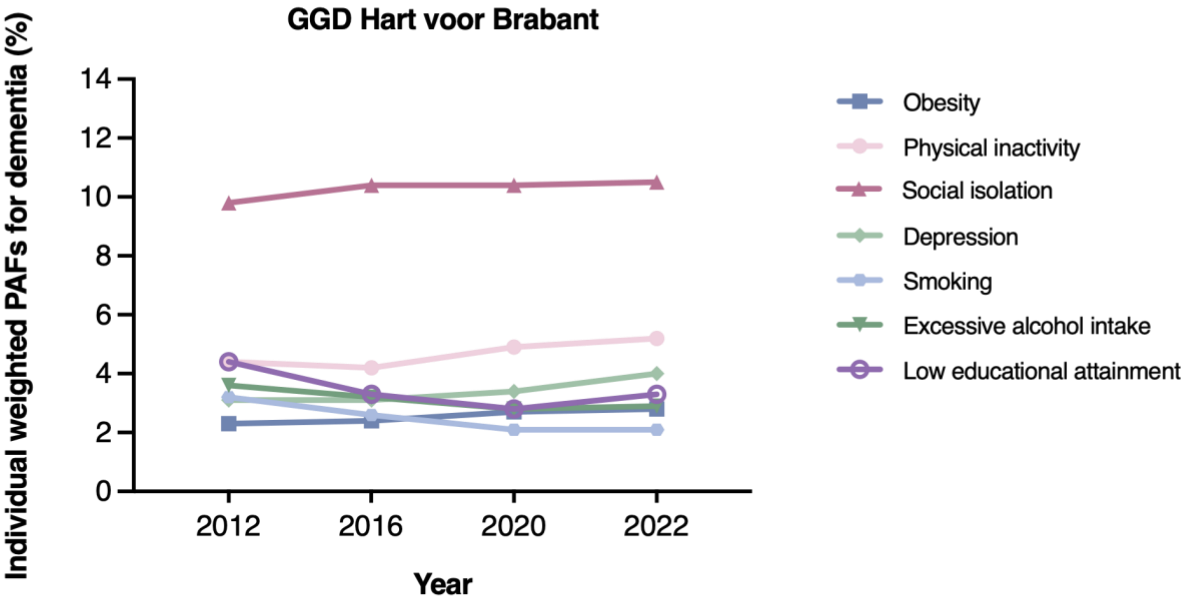

Figure S24. Individual weighted population attributable fractions of seven modifiable risk factors for dementia in municipal public health service (GGD) Hart voor Brabant in 2012, 2016, 2020, and 2022. ***Abbreviation: PAF: population attributable fraction.***

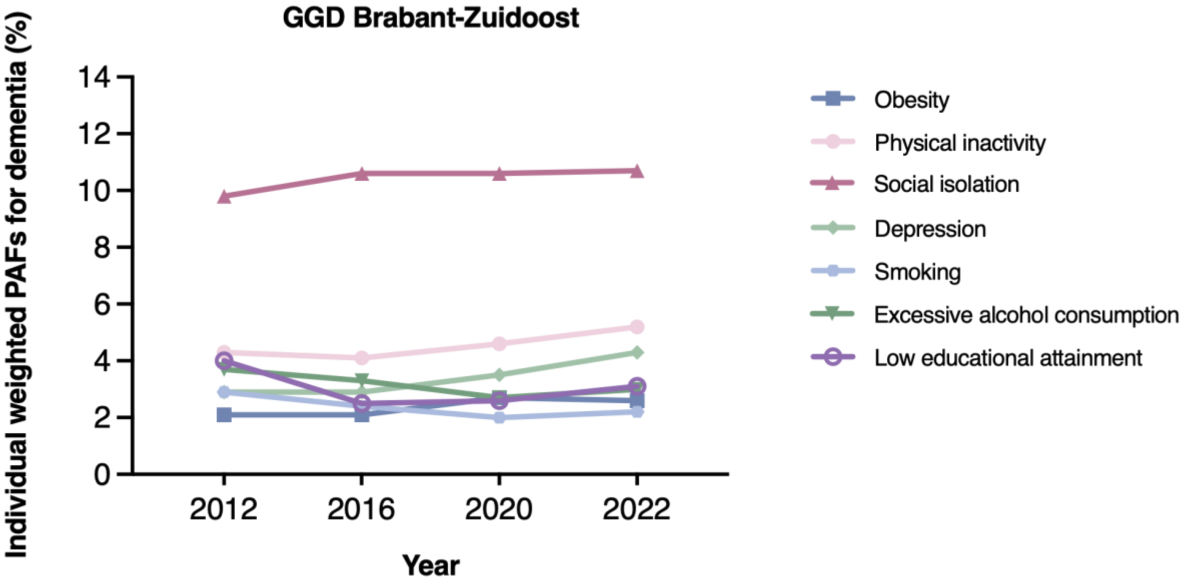

Figure S25. Individual weighted population attributable fractions of seven modifiable risk factors for dementia in municipal public health service (GGD) Brabant-Zuidoost in 2012, 2016, 2020, and 2022. ***Abbreviation: PAF: population attributable fraction.***

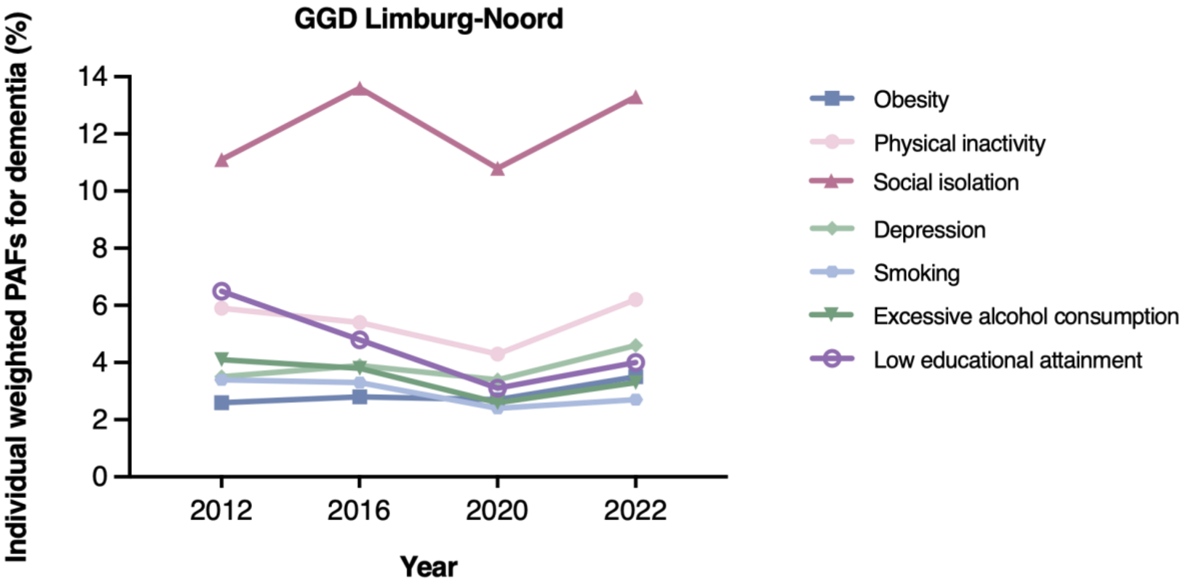

Figure S26. Individual weighted population attributable fractions of seven modifiable risk factors for dementia in municipal public health service (GGD) Limburg-Noord in 2012, 2016, 2020, and 2022. ***Abbreviation: PAF: population attributable fraction.***

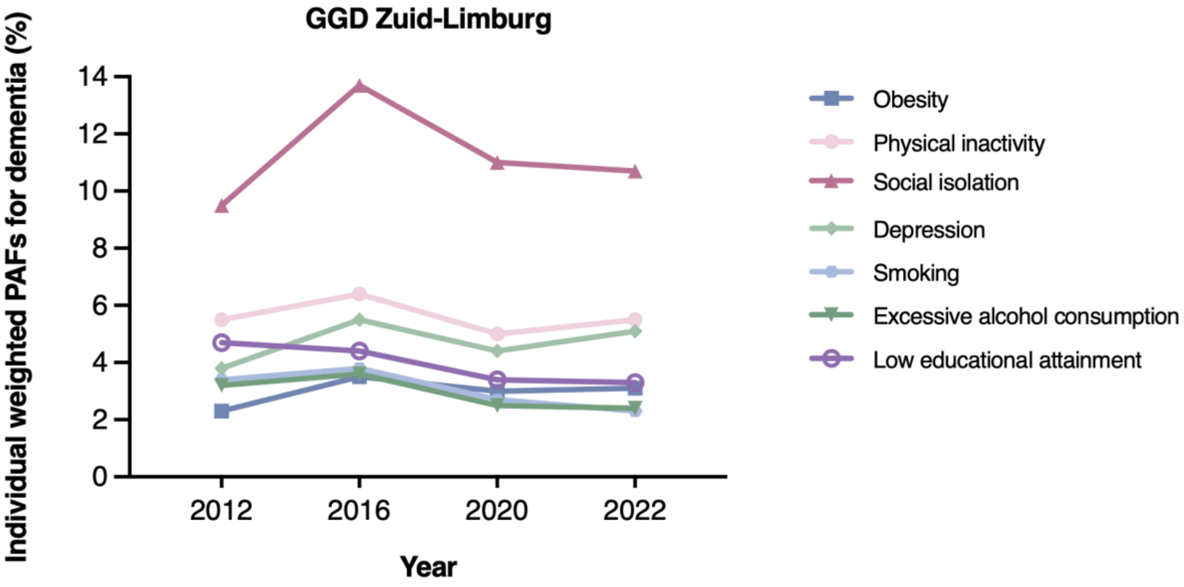

Figure S27. Individual weighted population attributable fractions of seven modifiable risk factors for dementia in municipal public health service (GGD) Zuid-Limburg in 2012, 2016, 2020, and 2022. ***Abbreviation: PAF: population attributable fraction.***

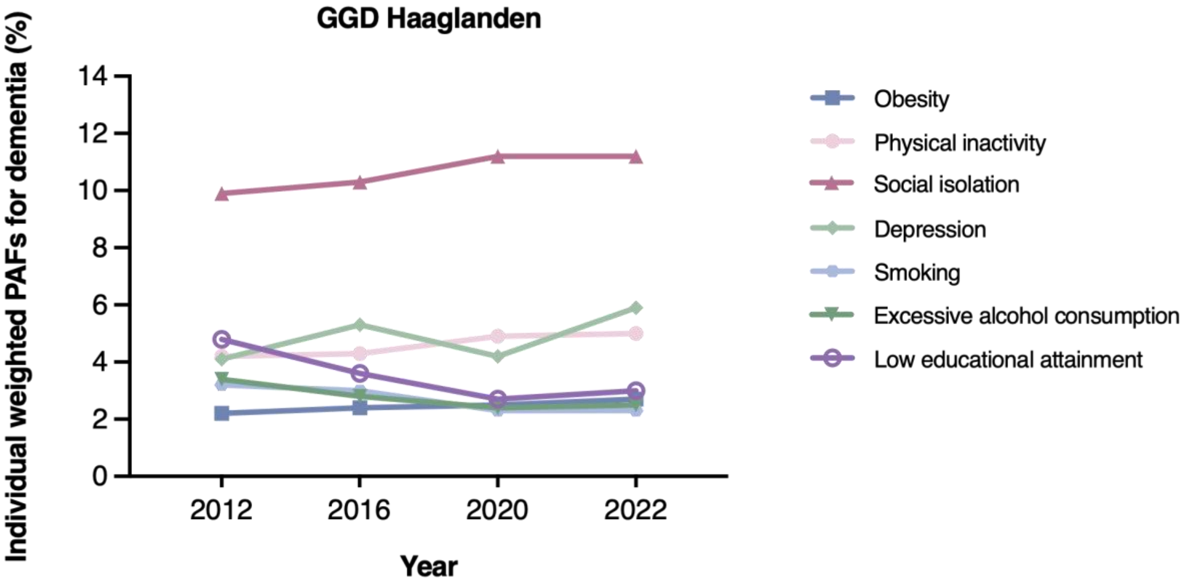

Figure S28. Individual weighted population attributable fractions of seven modifiable risk factors for dementia in municipal public health service (GGD) Haaglanden in 2012, 2016, 2020, and 2022. ***Abbreviation: PAF: population attributable fraction.***

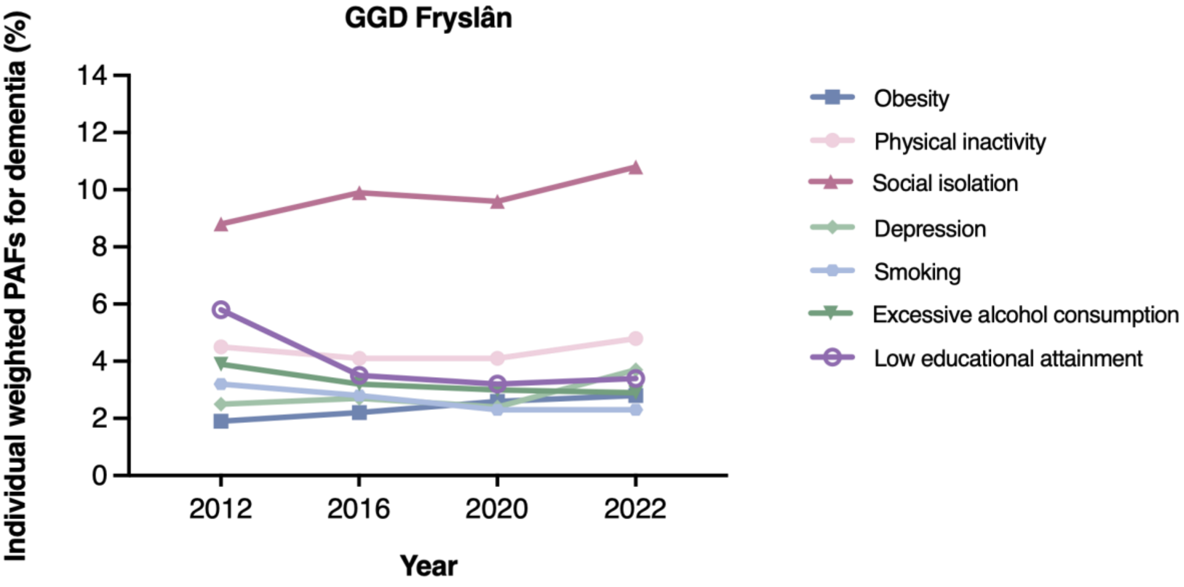

Figure S29. Individual weighted population attributable fractions of seven modifiable risk factors for dementia in municipal public health service (GGD) Fryslân in 2012, 2016, 2020, and 2022. ***Abbreviation: PAF: population attributable fraction.***

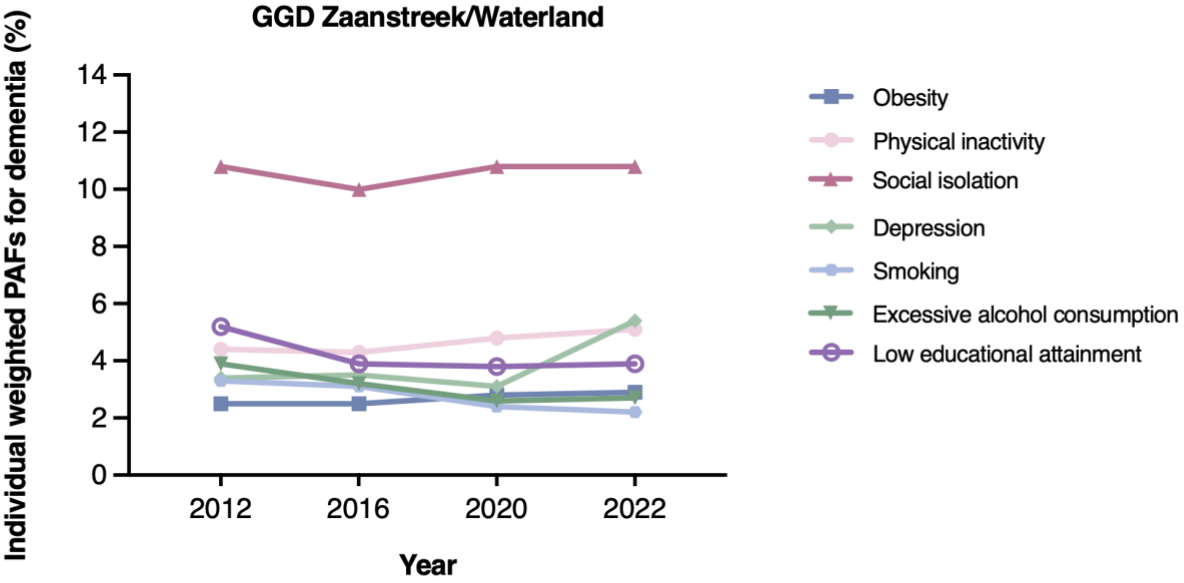

Figure S30. Individual weighted population attributable fractions of seven modifiable risk factors for dementia in municipal public health service (GGD) Zaanstreek/Waterland in 2012, 2016, 2020, and 2022. ***Abbreviation: PAF: population attributable fraction.***

Editorial note: the Lancet Group takes a neutral position with respect to territorial claims in published maps and institutional affiliations

References

1. Livingston G, Huntley J, Liu KY, et al. Dementia prevention, intervention, and care: 2024 report of the Lancet standing Commission. *The Lancet* 2024; **404**(10452): 572-628.
